# Triploidy—Observations in 154 Diandric Cases

**DOI:** 10.1371/journal.pone.0142545

**Published:** 2015-11-12

**Authors:** Nanna Brink Scholz, Lars Bolund, Mette Nyegaard, Louise Faaborg, Mette Warming Jørgensen, Helle Lund, Isa Niemann, Lone Sunde

**Affiliations:** 1 Department of Biomedicine, Aarhus University, Aarhus, Denmark; 2 Beijing Genomics Institute/HuaDa-Shenzhen, Shenzhen, China; 3 Department of Oncology, Aarhus University Hospital, Aarhus, Denmark; 4 Institute of Pathology, Aarhus University Hospital, Aarhus, Denmark; 5 Institute of Pathology, Aalborg University Hospital, Aalborg, Denmark; 6 Department of Gynecology and Obstetrics, Aarhus University Hospital, Aarhus, Denmark; 7 Department of Clinical Genetics, Aarhus University Hospital, Aarhus, Denmark; Hospital Authority, CHINA

## Abstract

Hydatidiform moles (HMs) are abnormal human pregnancies with vesicular chorionic villi, imposing two clinical challenges; miscarriage and a risk of gestational trophoblastic neoplasia (GTN). The parental type of most HMs are either diandric diploid (PP) or diandric triploid (PPM). We consecutively collected 154 triploid or near-triploid samples from conceptuses with vesicular chorionic villi. We used analysis of DNA markers and/or methylation sensitive-MLPA and collected data from registries and patients records. We performed whole genome SNP analysis of one case of twinning (PP+PM).In all 154 triploids or near-triploids we found two different paternal contributions to the genome (P1P2M). The ratios between the sex chromosomal constitutions XXX, XXY, and XYY were 5.7: 6.9: 1.0. No cases of GTN were observed. Our results corroborate that all triploid human conceptuses with vesicular chorionic villi have the parental type P1P2M. The sex chromosomal ratios suggest approximately equal frequencies of meiosis I and meiosis II errors with selection against the XYY conceptuses or a combination of dispermy, non-disjunction in meiosis I and meiosis II and selection against XYY conceptuses. Although single cases of GTN after a triploid HM have been reported, the results of this study combined with data from previous prospective studies estimate the risk of GTN after a triploid mole to 0% (95% CI: 0–1,4%).

## Introduction

Hydatidiform mole (HM) is an abnormal pregnancy characterized by vesicular swelling of the chorionic villi and hyperplasia of the trophoblastic layer [1]. Most HMs can be classified according to their histological features as either partial (PHM) or complete (CHM) or according to their genomic compositions as either diploid or triploid. Most often the parental type of PHMs is diandric triploids (PPM) and CHMs most often are diandric diploids (PP) [2]. Gestational trophoblastic neoplasia (GTN) is a complication to HM, and approximately 10% of patients with molar pregnancies subsequently are given chemotherapy to treat GTN [3]. The risk of GTN is lower after a PHM than after a CHM, and the risk is lower after a triploid HM than after a diploid HM; ploidy seemingly being a more reliable predictor of risk than morphology [4].

For several years diploid androgenetic moles were thought to result from fertilization of an “empty” oocyte [5]. In 2003 Golubovsky suggested a model of diploidization of dispermic triploids to explain several odd reproductive events [6]. We have previously reported eight cases of mosaicism consisting of one diploid androgenetic and one diploid biparental cell line (PP/PM) [7], and seven cases of twin pregnancies with a diploid androgenetic HM and a normal fetus (PP + PM) [8]. In three mosaics and in one case of twinning, the diploid androgenetic cells were homozygous and the genome was identical to the paternal genome in the diploid biparental cell line/twin conceptus, making it unlikely that these conceptuses resulted from dispermy. On the basis of these observations, we suggested that diploid androgenetic HMs in general originate in an oocyte with the maternal pronucleus retained, via a tri-pronuclear zygote and one or two abnormal cell divisions. In the homozygous diploid androgenetic HMs, the two paternal chromosome sets most likely originate from one spermatozoon, whereas in the more uncommon heterozygous diploid androgenetic HMs the two paternal chromosome sets could originate from one spermatozoon that was diploid due to a meiotic error; however most authors believe that they originate from two spermatozoa [7,9].

A triploid conceptus with two paternal contributions to the genome can in theory arise from either fertilization with a diploid spermatozoon or two different spermatozoa (dispermy). A diploid spermatozoon can arise from meiotic non-disjunction of entire genome sets or post-meiotic duplication of the haploid genome. Non-disjunction in the paternal meiosis I could result in a diploid sperm with the sex chromosomes XY, resulting in the combination XXY in the triploid conceptus. Non-disjunction in the paternal meiosis II could result in a diploid sperm with the sex-chromosomes XX or YY, resulting in the two combinations XXX or XYY in the triploid conceptuses, each type being equally frequent. Triploids caused by dispermy could show all three combinations: XXX, XXY and XYY, and theoretically the ratios between these should be 1:2:1. Post-meiotic duplication of the haploid genome in the paternal pronucleus should result in the combination XYY or XXX in the triploid conceptus. Thus, the ratio between the different sex chromosomal constitutions should depend on mechanisms in early development.

We studied a collection of 154 triploid conceptuses suspicious of HM, consecutively collected during a period of 25 years. Our aim was to determine the parental types, the ratio between the sex chromosomal constitutions XXX, XXY and XYY, and the risk of GTN. Our hypothesis of most diploid molar conceptuses originating in a zygote with three pronuclei of which two are generated from one spermatozoon was challenged. Therefore we performed whole genome SNP analysis in one previously published case of twinning PP + PM [7,8]. We previously found no case of GTN after 131 triploid moles [4]. In this study we present a follow up on this cohort and have included additional patients.

## Materials and Methods

### Study population

In the Danish Mole Project, established in April 1986, samples of fresh placental tissue from pregnancies clinically suspected of HM and parental blood samples are being collected. In this study, we have included samples which contained at least 10 vesicular chorionic villi each with a diameter of at least 1 mm and which showed triploidy or near-triploidy by karyotyping and/or flow cytometry. In the period April 1986–January 2012, 154 samples fulfilled these criteria. 94 samples were classified as triploid by karyotyping, 10 samples by flowcytometry and 50 samples were identified as triploid by both karyotyping and flowcytometry. Among the 144 karyotyped samples classified as triploid, 142 samples showed between 67 and 71 chromosomes and two samples showed a total of 73 and 77 chromosomes, respectively. In 26 cases we were in possession of both paternal and maternal blood samples. In 126 cases we had only a maternal blood sample and in 2 cases we had neither paternal nor maternal blood samples. Karyotypes and parental origin of the genome on 123 of these triploid cases have been published previously [10].

In addition to the 154 triploid cases included in this study, three samples forwarded to the Danish Mole Project that did not fulfill the inclusion criterion (at least 10 vesicular villi with a diameter of at least 1mm) were karyotyped and showed triploidy.

Further, one previously published case of twinning PP + PM (C0301 [8]) and parental blood samples were subjected to genome-wide SNP analysis.

We collected data from The Danish Cancer Registry for the period 1987–2009 and data from The Danish National Pathology Registry for the period 1987–2010 [11]. Furthermore, we collected data from the departments of oncology in Aarhus and Vejle where all patients from the western part of Denmark with GTN were treated with chemotherapy, for the period from 1987 to September 2011. 7 samples were collected from pregnancies terminated between May 2010 and January 2012. In order to obtain an adequate follow up on all cases, we searched the patient records in these 7 cases.

### DNA markers

The samples have been collected during the past 25 years and therefore different genotyping techniques have been used. Analyses of restrictions fragment length polymorphisms (RFLPs) were performed in the beginning of the study period [12], later analyses of microsatellite markers were performed. 36 samples were analyzed by RFLPs [12] and 118 samples were analyzed by AmpFlSTR® Identifiler® and/or AmpFlSTR® NGM SElect™. AmpFlSTR® Identifiler® covers the following 16 unlinked polymorphic loci; D8S1179, D21S11, D7S820, CSF1PO, D3S1358, TH01, D13S317, D16S539, D2S1338, Amelogenin, D5S818, FGA, D19S433, vWA, TPOX, D18S51. AmpFlSTR® NGM SElect™ covers the following 17 polymorphic loci; D3S1358, vWA, D16S539, D2S1338, D8S1179, D21S11, D18S51, D19S433, TH01, FGA, Amelogenin, D10S1248, D22S1045, D2S441, D1S1656, D12S391, SE33.

A conceptus was classified as having the parental type P1P2M if at least one locus that was fully informative, indicated this parental type (three different alleles of which two could be paternal, only, and one could be maternal, only) and the results for all other loci were consistent with this conclusion. The genome of the conceptus was also classified as having the parental type P1P2M if at least three loci showed two alleles of which one could only be paternal, the size of the signals indicating that there were two copies of this allele, at least one other locus showed three different alleles, and in at least three loci the signals indicated that if two of the three alleles were paternal there were two different paternal alleles. For cases where the results of the initial testing did not identify the parental type P1P2M, we tested further DNA markers. For cases showing aneuploidy, only loci on chromosomes showing trisomy were used for classification of the parental type.

For two cases we neither had a maternal nor a paternal blood sample (C0628, C0548). In these cases we determined the parental type of the genome using Methylation-Specific Multiplex Ligation-dependent Probe Amplification (MS-MLPA) [13] and AmpFlSTR®.

SNP array analysis was performed using 500 ng DNA from each sample and the Genome-Wide Human SNP Array 6.0 (SNP6) (Affymetrix, Santa Clara, CA, USA) according to manufacturer’s protocol. Genotypes were called using Genotyping Console software V4.1 and the Birdseed V2 algorithm (Affymetrix), resulting in a set of 904,569 markers. The genotyping call rate was above 95% for all samples. To filter out monomorphic markers in our dataset, we excluded SNPs with minor allele frequency of 0.1 or lower, resulting in a pruned dataset of 360,459 markers. The Identical By Descent (IBD) statistics and PI_HAT was calculated between pairs of samples using the 360,459 markers and the software PLINK [14]. Genomic positions are from the hg19 version of the human genome.

### Ethical approval

This project was approved by The Danish National Committee of Ethics in Science and registered in The Danish Data Protection Agency. All participants gave their written informed consent.

## Results

All of the 152 triploid or near-triploid cases analyzed along with a maternal sample, had the parental type P1P2M. The number of informative loci is given in Table 1. For 8 cases no locus was fully informative. In these cases we made the diagnosis P1P2M based on the criteria described in Methods and Materials (an average of 16 loci were analyzed per case). In 47 cases with only one fully informative locus we analyzed an average of 13 loci per case (ranging from 4 to 17 loci). In all but two of these 152 cases, the allelic composition was in accordance with P1P2M for all loci tested. In case C0213 the locus D18S51 was found with three different alleles of which only one was identical to a paternal allele and two were identical to the maternal alleles. In this case we analyzed five additional loci on chromosome 18 of which three loci showed two paternally inherited alleles and one maternal inherited allele and the results for the last two loci being in accordance with this parental type. Thus, we find it likely that the unusual allelic composition in D18S51 was due to a mutation, which has been observed for this locus before [15]. In C0700 the locus vWa was found with three different alleles of which none was identical to a maternal allele. The AmpFlSTR® NGM SElect™ kit analyzes another locus on chromosome 12, D12S391. The result in D12S391 was in accordance with the P1P2M parental type. C0700 could not have inherited three different alleles from the father and thus, we find it likely that a somatic mutation caused the odd observation in vWA, also previously observed [16].

**Table 1:** Number of fully informative loci in 152 (near) triploid cases with vesicular chorionic villi, analyzed along with a maternal sample.

Number of fully informative loci:	Number of cases with the given number of fully informative loci:
0	8
1	47
2	42
3	25
4	20
5	5
6	3
7	0
8	2

The two triploid cases for which we did not have a maternal sample (C0548 and C0628) were both found to have the parental type PPM by MS-MLPA analysis; and for both samples the AmpFlSTR analysis proved that there were 3 different alleles in two loci. Thus, the parental type of C0548 and C0628 was also classified as P1P2M.

Among the 144 karyotyped triploid or near-triploid cases, 57 had the sex chromosomal constitution XXX, 69 had the constitution XXY and 10 had the constitution XYY. Three cases had the more unusually constitutions; XX, XXXY and XXXX, respectively. In five cases the quality of the metaphases allowed determination of ploidy, whereas the sex chromosomal constitutions could not be determined. In all cases where the sex chromosomal constitution was established by karyotyping and the sex chromosomal loci were analyzed in the DNA marker analysis, consistent results were obtained. If we leave out the three cases with unusual sex chromosome constitutions and the five cases with unanalyzed sex chromosomes, the ratios XXX: XXY: XYY were 5.7: 6.9: 1.0 in the remaining 136 cases.

We found 0 cases of GTN in our cohort of 154 triploid P1P2M conceptuses. Minimal follow-up time was 6 months.

In addition to the 154 cases included in this study, three triploid samples forwarded to the Danish Mole Project did not fulfill the inclusion criterion. All three cases had the parental type P1P2M and none were followed by GTN. In two of these cases (C0411 and C0575) the karyotype was 69,XXX. Both were excluded from the project because no significant vesicles were seen in the sample forwarded for the project. The karyotype of the third case (C0886) was 69,XXY. This sample was excluded from the project because only 3 vesicles were seen.

**Fig 1:**
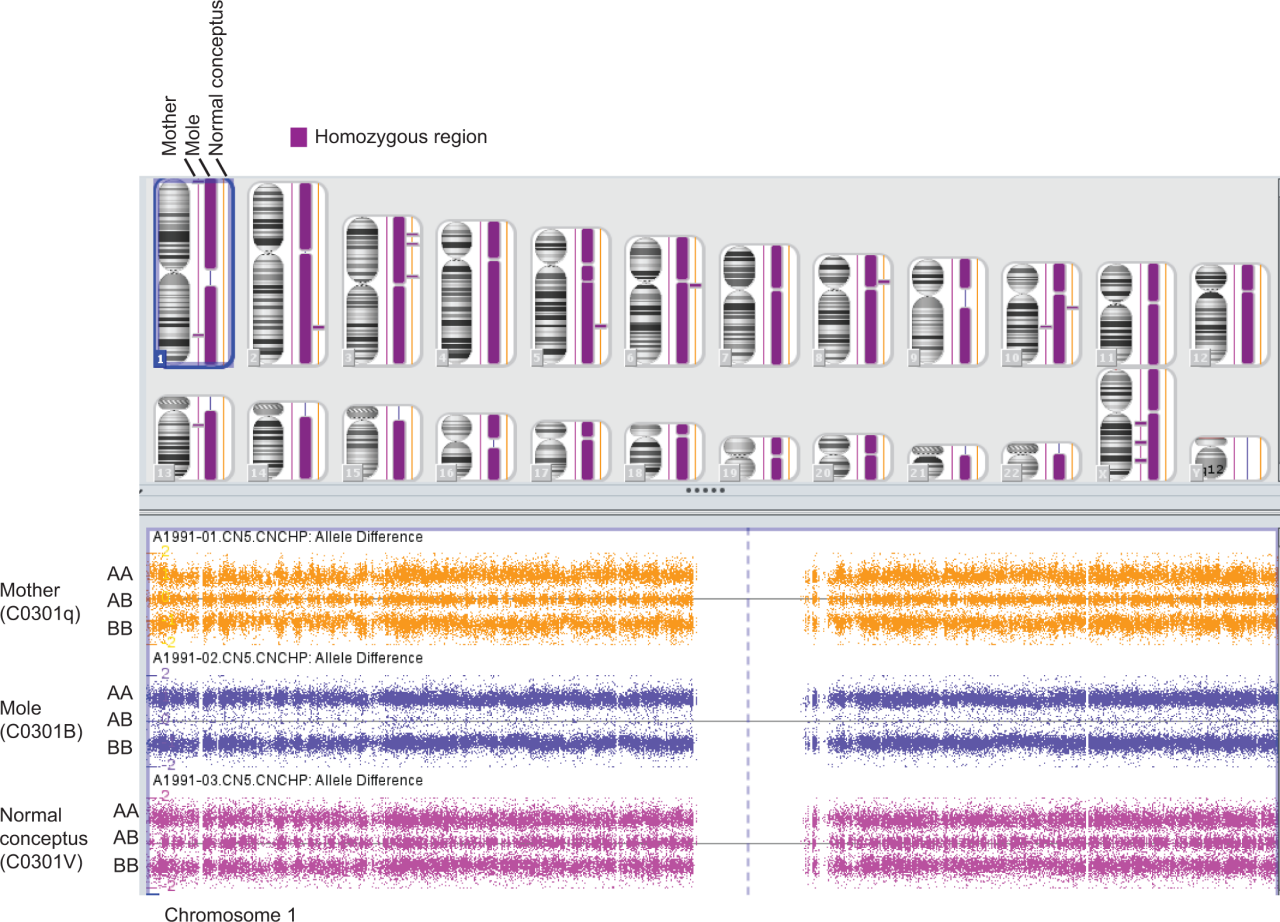
SNP6 array analysis, C0301. SNP6 array analysis of individual C0301q and her twin pregnancy (C0301B (mole, PP) and C0301V (normal conceptus, PM)) demonstrating that the molar pregnancy was homozygous for all markers across the autosomal chromosomes and the X chromosome. Upper panel: Regions with stretches of markers showing homozygousity are displayed in purple. Lower panel: Genotypes (AA, AB, and BB) on chromosomes 1 for the three samples, illustrating the general absence of markers with genotype AB for the mole pregnancy. Each dot represents a genotyped SNP.

Based on observations in HMs being part of twin conceptions and HMs being mosaics for diploid androgenetic and diploid biparental cell lines, we previously suggested that diploid moles originate in tri-pronuclear zygotes with two paternal genome sets, and that both of these frequently originate in one spermatozoon. Observation of heterozygosity for paternal markers in all the triploid conceptuses challenges this hypothesis. Therefore we performed genome wide SNP array of one of the previously analyzed twin pregnancies: the diploid androgenetic molar pregnancy (C0301B), the twin normal conceptus (C0301V) and the mother (C0301q) [8]. The analysis showed that the mole was homozygous for all markers across the genome (Fig 1). IBD calculations showed that the mole was not genetically related to the mother (PI_HAT = 0). The mother and the mole shared no regions identical by descent (Z1 = 0 and Z2 = 0) demonstrating a purely paternal origin of the genome for the mole (P1P1) (S1 Table). The mother and the normal conceptus, in contrast, shared one allele identical by descent for all markers (half of their genomes) as expected for parent-child pairs (S1 Table). Finally, the mole and the normal conceptus were found to share 1 allele IBD for all markers (half of their genomes), verifying that the paternal genome was identical in the normal conceptus and the mole. All together the SNP analysis verified that the mole was homozygous for the paternal genome, and that this genome was identical to the paternal genome carried by the twin.

## Discussion

HM is a rare condition. Denmark has a public health care system and the Danish Mole Project collects samples from all departments of gynecology and obstetrics in the western part of Denmark. In this study we analyzed all triploid cases from consecutively collected samples from conceptuses suspected of HM in the Danish Mole Project during 25 years. Investigating samples from this cohort has the advantage that all cases were collected from the same population, selected with a minimal bias, and analyzed systematically. As we did not use a histopathologic diagnosis of hydatidiform mole as an inclusion criterion, our data may not readily be comparable with observations in cohorts delineated by histopathological criteria [1]. However, as histologic diagnostics appear to be subject to inter- and intra-observer variability, our strategy may have eliminated one source of variability [17,18]. We have systematically included cases selected on a simple macroscopic criterion (the samples forwarded for the genetic laboratory showing at least 10 vesicles each with a diameter of 1mm or more). Furthermore, we genotyped three cases that had presented with a morphology that had raised suspicion of HM at the gynecological ward but did not fulfill the inclusion criteria. These all turned out to be diandric triploids with the parental type P1P2M. Thus, our inclusion criteria seem to be conservative. Analyzing DNA markers we only found one fully informative locus in 47 cases. This might be a weakness because individual loci can show aberrant results due to mutations as illustrated by our observation of unexpected allelic patterns in one locus in each of two cases. However, as we analysed an average of 13 loci in these 47 cases in all of which the results were in accordance with the conclusion P1P2M, it is unlikely that this is a significant source of error.

The parental types in triploid conceptuses suspected of HM have been investigated previously (Table 2).

**Table 2:** Parental types in triploid conceptuses with molar phenotype.

Study	Cohort from which the cases were selected	Number of diandric triploids	Method for genotyping	Parental type
Bifulco et al. 2008 [19]	Molar pregnancies and their morphologic mimics.	12	Genotyping, 15 loci, AmpFlSTR Identifiler PCR Amplification system.	P1P2M: 11/12. P1P1M: 1/12.
Murphy et al. 2009 [20]	PHMs.	8	Genotyping, 10 loci, AmpFlSTR Profiler kit.	P1P2M: 8/8.
McConnell et al. 2009 [21]	Products of conception specimens with any consideration of the possibility of a diagnosis of a HM.	7	Genotyping, 10 loci, AmpFlSTR Profiler kit.	P1P2M: 7/7.
Lipata et al. 2010 [22]	Products of conception with the possibility of molar pregnancy based on histology.	43	Genotyping, 15 loci, AmpFlSTR Identifiler PCR Amplification system.	P1P2M: 42/43. P1P1M: 1/43.
Buza et al. 2013 [18]	Early abortions suspicious of molar gestation.	56	Genotyping, 15 loci, AmpFlSTR Identifiler	P1P2M: 55/56.P1P1M: 1/56.
Present study	Conceptuses suspected to be hydatidiform moles by the gynecologist and showing ≥ 10 vesicular villi with a diameter of ≥ 1 mm.	154	Genotyping using RFLPs and/or microsatellite loci, analyzing further loci until heterozygosity for paternal markers was identified.	P1P2M: 154/154.

Murphy et al. [20] and McConnell et al. [21] analyzed small numbers of conceptuses and found, as we did, the parental type P1P2M in all cases. Lipata et al. [22], Buza et al. [18], and Bifulco et al. [19] investigated larger numbers of triploid conceptuses with molar phenotypes and reported the P1P2M parental type in most cases, but each reported data on a single case suggestive of homozygosity for one paternal genome set (P1P1M). However, the P1P1M parental type is a diagnosis of exclusion and the validity is dependent on the number of investigated loci. Thus, it is possible that the cases reported to be homozygous for paternal markers would have turned out to be heterozygous if additional loci had been analyzed.

We observed the ratios between cases with the sex chromosome constitutions XXX, XXY, and XYY: 5.7: 6.9: 1.0. Thus the XYY combination was rare compared to XXY and XXX. A low frequency of XYY conceptuses in diandric triploids has been observed by others, as well [23–26]. McWeeney et al. [23] found the frequency of conceptuses with the constitution XYY to be significantly higher before implantation compared to the frequency in clinical pregnancies. This observation suggests that a conceptus with the sex chromosome constitution XYY is less likely to survive than a conceptus with the sex chromosome constitution XXX or XXY.

Diandric triploids caused by post-meiotic errors should have the parental type P1P1M which we did not find in any cases. Thus, post-meiotic errors are unlikely to cause triploid HMs. 73 triploid cases had the sex chromosomes XXY. This combination cannot result from a meiosis II error. 67 cases had the sex chromosomes XXX and 11 had XYY. These two combinations cannot be caused by paternal meiosis I errors. Thus, neither errors in meiosis I nor II can be the only mechanism causing triploid HMs. Also, dispermy seems unlikely as the only mechanism: Even if we assume selection against conceptuses with the XYY combination, the fact that conceptuses with the XXX and XXY constitutions were almost equally frequent, conflicts with the expected ratio of 1:2 in dispermy (χ^2^: p = 0.0003). The ratios we observed could be due to approximately equal frequencies of meiosis I and meiosis II errors with selection against the XYY conceptuses or a combination of dispermy, non-disjunction in meiosis I and meiosis II and selection against the XYY conceptuses.

However, in the early literature the overall agreement favors that the majority of diandric triploids results from dispermy [26,27]. Further, by including pericentromeric markers, Zaragoza et al. [28] found indications of dispermy in 37 of 43 triploid diandric conceptuses whereas four cases seemed to be caused by errors in meiosis I, one case by an error in meiosis II, and one case by a postmeiotic mitotic error; and McFadden et al. [29] found indications of dispermy in all of 14 diandric triploids. If we assume that dispermy accounts for most of our cases, we either observe an excess of conceptuses with the constitution XXX or a deficit of conceptuses with the XXY constitution. An “excess” of conceptuses with the constitution XXX could be due to some cases being caused by meiosis II errors. Another possibility is selection against conceptuses with the XXY constitution, in line with (but in a minor scale) the suggested selection against conceptuses with the combination XYY. McWeeney et al. found a tendency for a decrease in the proportion of conceptuses with XXY compared to XXX [23], corroborating this possibility. However, the true explanation of the frequencies of the various karyotypes in triploid diandric conceptuses is not yet known.

The results from the present study challenge our hypothesis of most diploid molar conceptuses originating in a zygote with three pronuclei of which two are generated from one spermatozoon [7]: Among 154 diandric triploids with molar morphology we found no case of P1P1M triploidy which is significantly different from our previous observation of four cases with seemingly only one paternal genomic contribution among 15 cases of mosaics or twinning [7,8] (p<0.0001, Fisher’s exact test). In the following we discuss possible explanations of why we did not find any P1P1M triploids in our cohort of diandric triploid conceptuses with molar morphology but previously found mosaics and twins with the parental types P1P1/P1M and P1P1 + P1M.

**Fig 2:**
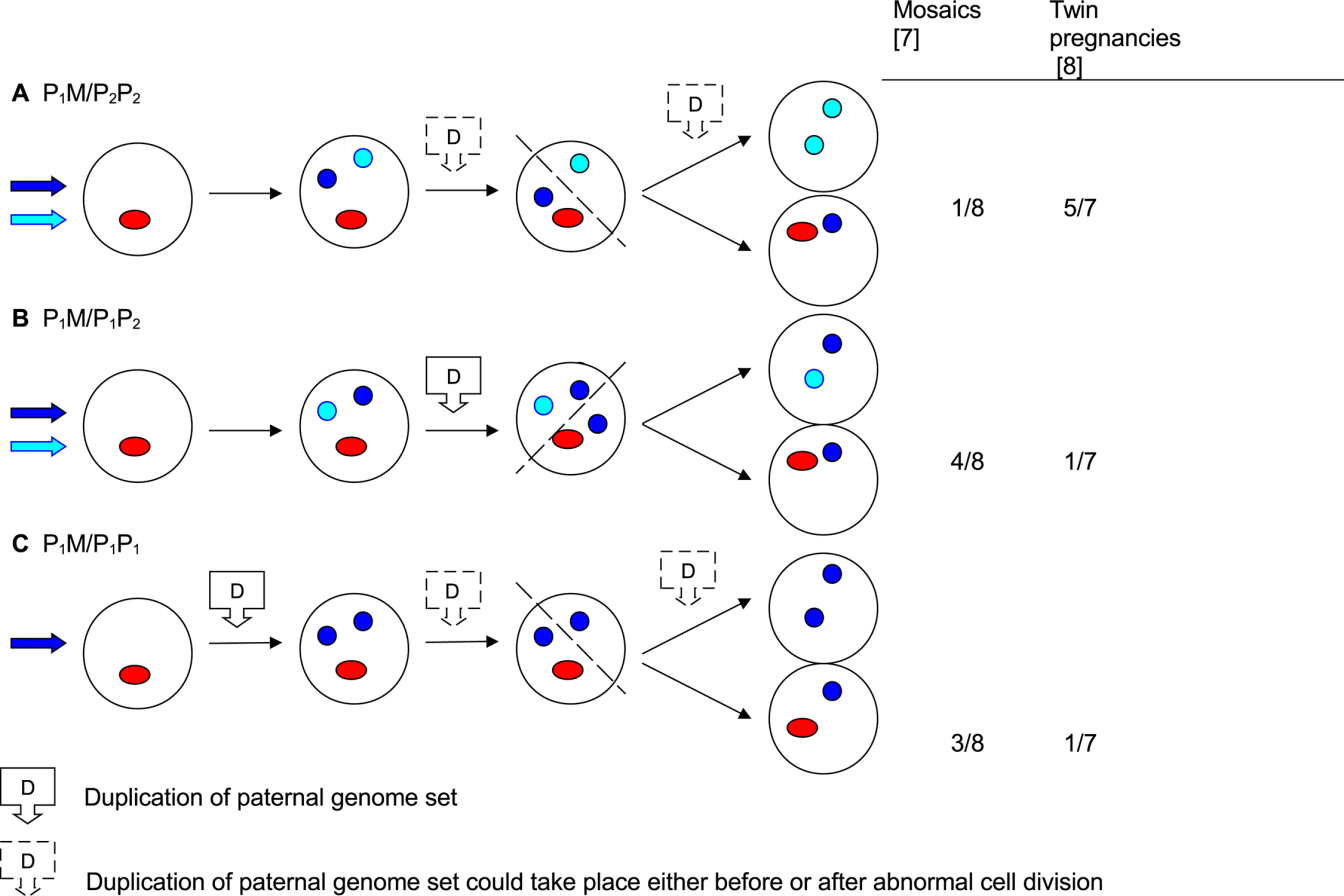
reproduced [7]. Possible fertilizations, endoreduplications, and abnormal cell divisions in mosaic hydatidiform moles (HMs), and twin gestations including an HM. (a) Fertilisation by two sperms; one giving rise to the paternal genome set in the diploid biparental cell population, the other giving rise to both genome sets in the diploid androgenetic cell population via endoreduplication. (b) Fertilisation by two sperms; one giving rise to one of the paternal genome sets in the diploid androgenetic cell population, the other contributing one genome set to both cell populations via endoreduplication. (c) Fertilisation by one sperm that via two endoreduplications gives rise to three identical paternal genome sets, of which two constitute the genome of the diploid androgenetic cell population and one is the paternal genome set in the diploid biparental cell population.

1. Not all diandric triploids present as HMs [10]. It has been suggested that only one-half of diandric triploids are HMs [28]. If this is true, conceptuses with the parental type P1P1M might not develop the classic molar phenotype. However, it is not obvious why the P1P1M parental type would never result in a HM phenotype, when taking into account that diploid HMs most often have the parental type P1P1 [30]. Furthermore, the fact that we discovered the parental type P1P2M in all of 3 triploid conceptuses with a phenotype too inconspicuous to be included in The Danish Mole Project makes this explanation less likely.
2. It may be argued that P1P1/P1M mosaics and P1P1+P1M twins do not exist. As homozygosity is a diagnosis of exclusion, one could imagine that the molar part of the mosaics and twins classified as having homozygous paternal genomes might have shown paternal heterozygosity if additional loci had been analyzed. However, Makrydimas et al. [31] reported a case of confined placental mosaicism with identical paternal homozygous/hemizygous contribution to both the HM and the fetus, identified by analyzing both centromeric and distal markers and we identified identical paternal markers genome-wide in one case of twinning between a diploid HM and a normal conceptus, indicating that this mechanism do exist.
3. In our previously published hypothesis we suggest that most molar conceptuses originate in an oocyte fertilized by either one or two spermatozoa (Fig 2, reproduced [7]). Fertilization by two spermatozoa could result in a P1P2M tri-pronuclear stadium whereas fertilization by one spermatozoon could result in a P1P1M tri-pronuclear stadium. The latter is the only one, in which duplication of the paternal genome set should happen before, and not after the tri-pronuclear stadium. Maybe this first duplication is the beginning of some kind of a cascade where the first duplication (prior to the P1P1M stadium) is always followed by a second duplication. The fact that the paternal genome set is duplicated in a zygote with one maternal and one paternal pronucleus suggests that this cell is subject to some kind of abnormality. The same abnormality could possibly prevent the cell from staying in the P1P1M stadium, increasing the chance of diploidization.
4. Functional centrioles are only inherited from the father [32]. Thus, after fertilization with one spermatozoon, one would expect a normal number of centrioles and after dispermy one would expect two pairs of centrioles. Centrioles are important for cell divisions and one could imagine that a tri-pronuclear zygote with a normal number of centrioles (fertilization by one spermatozoon) acts differently in the first cell division compared to a tri-pronuclear zygote with two pairs of centrioles (fertilization by two spermatozoa). This difference could cause that P1P1M tri-pronuclear zygotes always undergo diploidization and P1P2M tri-pronulear zygotes are more likely to turn into a triploid conceptus.

Thus, fertilization of an oocyte with the maternal pronucleus retained, by one or more spermatozoa, possibly followed by duplication of a paternal pronucleus, and followed by an abnormal cell division is still a plausible mechanism for the origin of diploid androgenetic conceptuses. There are two differences between a P1P1M zygote and a P1P2M zygote. One is the fact that only in a P1P1M zygote, duplication of one genome set has taken place. The other difference is the number of centrioles. We suggest that one of these differences hold the explanation to why zygotes with two identical pronuclei rarely, if ever lead to triploidy. It would be interesting to study the first cell divisions in zygotes that lead to twinning with a normal conceptus and a HM, or a molar mosaic. Although such pregnancies are rare, the advent of the embryoscope has already allowed photographing embryons with time lapses [33] and leaves hope that further refinements will allow filming or at least more frequently picturing of many zygotes during their first cell divisions.

HM may be regarded as a pre-malignant disorder, as GTN may follow. The frequency of GTN after CHM has been reported to be 24,2% by Kang et al. [34]. The risk after PHM has been estimated to be 0.2–5.6% [9]. By extending our previous studies [4] we found no cases of GTN in 154 patients with triploid diandric conceptuses with molar morphology, corroborating the results of previous prospective studies: Kaneki et al. reported no cases of GTN in 20 cases of triploid diandric conceptuses with molar morphology [35], Ohama et al. reported no cases of GTN in 47 triploid HMs and Lawler et al. reported no cases of GTN in 44 triploid HMs [2,36]. Thus, among prospectively studied cases, the frequency of GTN after triploid moles/diandric triploids is 0/265, giving an estimated risk of 0% (95% CI: 0–1.4%). However, Seckl et al. reported 3 cases of choriocarcinoma after triploid PHMs [37] and Cheung et al. reported 2 cases of metastatic GTN after triploid PHMs [38], illustrating that although the risk is low, GTN can occur after a triploid HM.

Traditionally HMs are classified according to the phenotype, and the risk of GTN after PHM and CHM have been determined several times. However, poor inter- and intraobserver agreement makes it clear that it is not easy to distinguish partial and complete HMs [17,18]. Lately, genetic methods have been used in the diagnostics and classification of HMs. It has been suggested that diandric triploids are called PHM and diploid HMs are called CHM [19,22]. We suggest that a consistent nomenclature is used as the risk after a PHM does not equal the risk after a triploid HM. When classifying HMs the nomenclature should correlate to the methods used for classification. We suggest that HMs classified by morphological methods should be termed “partial” or “complete” (the phenotype) and that HMs diagnosed by genetic methods should be referred to for instance “diploid”, “triploid”, "diploid androgenetic", "diploid biparental" etc. dependent on which information is available (the genotype).

## Supporting Information

S1 File Related publication [10]. (PDF)

S2 File Related publication [4]. (PDF)

S1 Table Genetic relatedness between pairs of individuals assessed by the PI_HAT method, which estimates the proportion of the genomic variation shared identical by descent (IBD). Identical twins are 100% identical by descent (PI_HAT = 1.0), first-degree relatives are on average 50% IBD (PI_HAT = 0.5), second-degree relatives are on average 25% IBD (PI_HAT = 0.25). For each comparison, Z2, Z1 and Z0 are the proportion of markers showing 2, 1 and 0 alleles identical by descent, respectively. The IDB proportions and PI_HAT showed that the mother has no genetic relationship with the molar pregnancy. The normal conceptus and the mole were related as parent-child (and not as siblings) because they share the paternal genome identical by descent.
(DOCX)
