# Supplementary material for: Triploidy—Observations in 154 Diandric Cases
Source: PLoS One. 2015 Nov 12;10(11):e0142545. doi: 10.1371/journal.pone.0142545 (PMC4642992; doi:10.1371/journal.pone.0142545)
Supplement: S1 File — (PDF) [file pone.0142545.s001.pdf]

OBSTETRICS

Triploid pregnancies: genetic and clinical features of 158 cases

Mette W. Joergensen, MD; Isa Niemann, PhD; Anders A. Rasmussen, PhD; Johnny Hindkjaer, MSc; Inge Agerholm, PhD; Lars Bolund, DMSc, MD; Lone Sunde, PhD

OBJECTIVE: The purpose of this study was to analyze the correlation between the genetic constitution and the phenotype in triploid pregnancies.

STUDY DESIGN: One hundred fifty-eight triploid pregnancies were identified in hospitals in Western Denmark from April 1986 to April 2010. Clinical data and karyotypes were collected retrospectively, and archived samples were retrieved. The parental origin of the genome, either double paternal contribution (PPM) or double maternal contribution (MMP) was determined by an analysis of methylation levels at imprinted sites.

RESULTS: There were significantly more PPM than MMP cases ($P <$ .01). In MMP cases, the possible karyotypes had similar frequencies, whereas, in PPM cases, 43% had the karyotype 69,XXX, 51% had the karyotype 69,XXY, and 6% had the karyotype 69,XYY. Molar phenotype was seen only in PPM cases. However, PPM cases with a nonmolar phenotype were also seen. For both parental genotypes, various fetal phenotypes were seen at autopsy. Levels of human chorionic gonadotropin in maternal serum were low in MMP cases and varying in PPM cases, some being as low as in the MMP cases.

CONCLUSION: In a triploid pregnancy, suspicion of hydatidiform mole at ultrasound scanning, by macroscopic inspection of the evacuated tissue, at histology, or because of a high human chorionic gonadotropin in maternal serum level each predict the parental type PPM with a very high specificity. In contrast, the sensitivity of these observations was <100%.

Key words: diagnosis, genomic, human chorionic gonadotropin, hydatidiform mole, triploidy

Cite this article as: Joergensen MW, Niemann I, Rasmussen AA, et al. Triploid pregnancies: genetic and clinical features of 158 cases. Am J Obstet Gynecol 2014;211:370.e1-19.

Triploidy, which is one of the most common chromosome aberrations, is estimated to occur in 1-2% of all human conceptions.[1]

Triploid pregnancies can be classified according to the genetic constitution. The genome in a triploid pregnancy is either digynic, which consists of 2 maternal and 1 paternal set of chromosomes (MMP), or diandric, which consists of 2 paternal and 1 maternal set of chromosomes (PPM).[1] Extremely varying frequencies of the 2 parental types have been reported (the frequency of PPM cases ranges from 20—73%).[1-9]

Triploid pregnancies can also be classified according to their phenotype. By the use of ultrasound scanning (US), it has been found that the placenta can appear normal, small, or enlarged and with or without vesicular changes. If a fetus is present, it can exhibit malformations but can also appear normal. Growth restriction appears to be a common, but not a consistent, phenomenon.[10] In studies that have been focused on levels of serum maternal human chorionic gonadotropin (MS-hCG), low, normal, and high levels have been reported.[11-13] In some studies that involved postmortem examinations of triploid pregnancies, 2 distinctive phenotypes have been suggested: type 1, relatively well-grown fetuses with proportionately sized body parts and large placentas with the morphologic condition of a partial hydatidiform mole; type 2, fetuses with severe growth retardation, an uneven development of various body parts that typically results in a relative macrocephaly and small, noncystic placentas.[4,14]

However, a systematic description and classification of a large cohort of triploid pregnancies has not been published. We correlate the parental origin of the genome with karyotype and phenotype of the triploid pregnancy and with the concentration of MS-hCG in a series of 158 triploid pregnancies.

From the Department of Clinical Genetics, Vejle Hospital, Vejle (Drs Joergensen and Rasmussen); Institute of Regional Health Research, University of Southern Denmark, Odense (Dr Joergensen); Department of Gynecology and Obstetrics, Aarhus University Hospital (Dr Niemann); Department of Biomedicine, Aarhus University (Drs Bolund and Sunde); Department of Clinical Genetics, Aarhus University Hospital (Dr Sunde); the Fertility Clinic and Center for Preimplantation Genetic Diagnosis (Mr Hindkjaer), Aarhus University Hospital, Aarhus; and the Fertility Clinic, Horsens Hospital, Horsens (Dr Agerholm), Denmark.

Received Nov. 25, 2013; revised Feb. 10, 2014; accepted March 14, 2014.

Supported by Vejle Hospital, University of Southern Denmark, Aase and Einar Danielsen Foundation, the Augustinus Foundation, the Medical Research Foundation of Central Region Denmark, and the A. P. Moeller and Chastine McKinney Moeller Foundation.

The authors report no conflict of interest.

Reprints: Mette W. Joergensen, MD, Department of Clinical Genetics, Vejle Hospital, Kabbeltoft 25, 7100 Vejle, Denmark. mette.warming.joergensen@rsyd.dk

0002-9378/$36.00 • © 2014 Elsevier Inc. All rights reserved. • http://dx.doi.org/10.1016/j.ajog.2014.03.039

MATERIALS AND METHODS

The study was approved by the Regional Committees on Health Research Ethics in Southern Denmark and the Central Region Denmark and by the Danish Data Protection Agency.

We aimed at including all triploid pregnancies in Western Denmark (Jutland) that were diagnosed from April 1986 to April 2010. In this period, triploid pregnancies were registered either in The Danish Mole Project (DMP), The Danish Cytogenetic Central Registry (DCCR), or both.

As part of the DMP, gynecologists in Jutland forward fresh placental tissue from pregnancies that are suspected to be hydatidiform moles. Triploidy is identified by karyotyping uncultured and/or cultured cells and/or by measurement of the nuclear DNA contents by flow cytometry of unfixed nuclei with the use of 2 external controls.[15]

The DCCR has held data on all karyotypes that have been made for clinical purposes in Denmark since 1960. All women in Jutland who have been registered in the DCCR with a triploid pregnancy in the study period were identified. With the use of the women's Civil Registration System number, their place of residence was found, and the laboratory that performed the genetic analysis was identified.

Frozen cell cultures and/or DNA were retrieved from the departments of clinical genetics at Aarhus University Hospital and Vejle Hospital and from the DMP.

Determination of the parental origin of the genome

DNA was purified with QIAamp DNA Mini Kit (Qiagen, Hilden, Germany). The triploid samples were analyzed with methylation-specific multiplex ligation–dependent probe amplification.[16] In brief, the level of methylation at 2 imprinted loci in the 11p15 region was measured with the SALSA MLPA ME030 BWS/RSS probemix (Kit ID MRCH 30808 and MRCH 36554; MRC-Holland, The Netherlands). Because imprinted loci are methylated in a parent-of-origin–specific manner, we previously have shown that the methylation level is an indicator of the proportion of maternally vs paternally inherited genomic material.[16]

TABLE 1
Karyotypes correlated to parental type

Karyotype	Total, n	Double paternal contribution/double maternal contribution, n
69,XXX	61	49/12
69,XXY	69	59/10
69,XYY	7	7/0
Total	137	115/22

Joergensen. Classification of triploid pregnancies. Am J Obstet Gynecol 2014.

Clinical data

Information regarding the first day of last menstrual period, the maternal serum concentration of the beta subunit of human chorionic gonadotropin (MS-beta hCG; if tested in the first trimester), the maternal serum concentration of total human chorionic gonadotropin (MS-total hCG; if tested later in pregnancy), and the results from histopathologic examination/autopsy of the pregnancy were retrieved from medical records. Data on the morphologic findings that were observed by US were retrieved from Astraia (a database used by the Danish Departments of Gynecology and Obstetrics).

Gestational age was calculated from last menstrual period (GA_{LMP}) by Naegele's rule.[17]

Classifications and definitions

The placenta was categorized as molar if the sonographer described the placenta with cystic spaces or molar appearance, if the placenta fulfilled the criteria for inclusion in the DMP, or if a histopathologic diagnosis of hydatidiform mole was made.

Fetal abnormalities detected by US were defined as any fetal malformation and/or oligohydramnios and/or intrauterine growth restriction (IUGR). With regards to abnormalities of the fetus that were found after termination, the morphologic condition that was described by the obstetrician after evacuation, and the autopsy findings were retrieved.

For the MS-beta hCG and MS-total hCG, the multiples of the median (MoMs) were calculated by expressing the absolute concentrations relative to the median for the relevant GA_{LMP}. For MS-beta hCG, medians were calculated by the algorithm described by Spencer.[18] For MS-total hCG, medians were calculated by the exponential regression.[19] Low levels of MS-hCG were defined as <0.5 MoM; high levels were defined as >3.0 MoM.

Categoric variables were compared with the use of the chi-square test, Fisher's exact test (Microsoft Excel 2010; Microsoft Corporation, Redmond, WA), or unpaired *t*-test (Graph Pad Prism, version 5.01; GraphPad Software, Inc, La Jolla, CA). GA_{LMP}s were compared with the use of a 2-tailed Mann-Whitney test (Graph Pad Prism software version 5.01; GraphPad Software, Inc). Significance level was set at a probability value of $< .05$.

RESULTS

Data on each triploid pregnancy can be seen in the Appendix (Supplementary Table).

A total of 183 triploid pregnancies had been registered: 140 in the DMP, 41 in the DCCR, and 2 who were registered in both the DMP and the DCCR. We succeeded in retrieving samples from a total of 158 cases: 121 cases from the DMP, 35 from the DCCR, and the 2 cases who were registered both in the DMP and the DCCR. Clinical data were available in all 158 cases.

GA_{LMP} at termination was available in 115 cases (21 MMP and 94 PPM) and ranged from 10 + 5 to 30 + 3 weeks'

TABLE 2
Autopsy findings in 15 triploid pregnancies

Identification no.	Parental type	GA_{LMP} at termination in wk + d	Histopathologic morphologic condition of placenta	Histopathologic observations in fetus
70	PPM	12 + 2	Normal	Intrauterine growth restriction; no external malformations
40	PPM	13 + 6	Pregnancy product	CRL, 7 cm
71	PPM	18 + 3	Normal	No external malformations, renal cysts bilaterally, dysplasia of the right kidney, and possible hypoplasia of the thymus
77	PPM	19	Partial mole	CRL, 12 cm; feet, 20 mm, which indicated gestational age at 16 weeks; sacral skin defect 8 mm; low set ears; bifid uvula; hyperplasia of Leydig cells, proliferation of adrenal cord
120	PPM	20 + 1	Partial mole	Total length, 19 cm; feet, 20 mm; cleft lip and palate
81	PPM	22	Partial mole	CRL, 3.5 cm; weight, 3.5 g; long body small head; adhesion of left upper arm to facial structures; adhesion between upper and lower leg; agenesis of hands and feet; anus abnormally placed in columnar; organs degenerated
122	PPM	24 + 5	Partial mole	Intrauterine growth restriction and deformities of feet (not specified)
123	PPM	26 + 2	Partial mole	Syndactyly of 3th and 4th finger and toe bilaterally and micro-penis; pulmonary hypoplasia; multicystic renal dysplasia; atresia of proximal ureters
23	MMP	30 + 3	Normal	Square-shaped cranium; low-set ears; narrow nose; syndactyly of the 3th and 4th finger on the left hand and of the 4th and 5th toe on both feet; migration defect in cerebrum; atrophic thymus; and perforated small intestine
18	MMP	15 + 2	Normal	Asymmetric growth retardation; low-set ears; increased distance between eyes; micrognathia, syndactyly of 3th and 4th fingers on the left hand and 2th and 3th toe on the right foot
19	MMP	15 + 3	Normal	No external malformations
10	MMP	16 + 1	NA	Asymmetric growth retardation[a]
12	MMP	16 + 6	NA	Small of date possibly anencephalic[a]
14	MMP	18 + 4	NA	Large head; low-set ears; cleft palate; myelomeningocele over spinal column; omphalocele[a]
21	MMP	20 + 5	Normal	Hydrocephaly; micrognathia; syndactyly of 3th and 4th finger on the left side and 3th and 4th toe on the right side; possible hypoplasia of the lungs

CRL, crown rump length; GA_{LMP}, gestational age calculated from last menstrual period; *MMP*, double maternal contribution; *NA*, not available; *PPM*, double paternal contribution.

[a] Morphologic condition described by the obstetrician.

Joergensen. Classification of triploid pregnancies. Am J Obstet Gynecol 2014.

gestation (median, 16 + 6 weeks). The difference between the median GA_{LMP} at termination for PPM and MMP cases (17 + 0 and 15 + 5 weeks gestation, respectively) was not significant ($P = .7$).

Parental origin of the genome

One hundred thirty-four of the 158 included cases (85%) had the parental type PPM (all of the 121 cases from the DMP, 11 cases from the DCCR, and the 2 cases registered in both registers), and 24 cases (15%) had the parental type MMP (all 24 were found in the DCCR). The difference between the 2 proportions was statistically significant ($P < .01$).

FIGURE
Maternal human chorionic gonadotropin levels

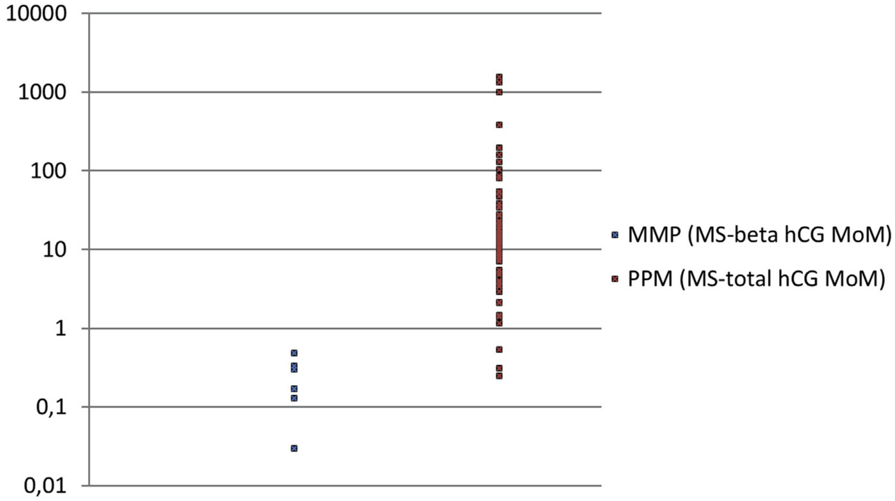

Data are given in multiples of the median (*MoM*) according to parental type.

MMP, double maternal contribution; *MS-beta HCG*, maternal human chorionic gonadotropin level; *PPM*, double paternal contribution.

Joergensen. Classification of triploid pregnancies. Am J Obstet Gynecol 2014.

The parental type of 105 cases (103 PPM and 2 MMP) has been presented previously.[16]

Karyotype

A karyotype was registered in 137 cases (Table 1): among the 115 cases with the parental type PPM, 49 cases (43%) had the karyotype, 69,XXX; 59 cases (51%) had the karyotype 69,XXY, and 7 cases (6%) had the karyotype 69,XYY. All cases with the karyotype 69,XYY were classified as hydatidiform moles by the histopathologist.

Among the 22 cases with the parental type MMP, 12 cases (55%) had the karyotype 69,XXX, and 10 cases (45%) had the karyotype 69,XXY.

Placenta morphologic findings

Suspicion of a molar pregnancy based on US was raised in 75 of 103 PPM cases, but in none of 22 MMP cases. All cases included in the DMP had the parental type PPM. The reports from histopathologic examination of the placentas were available in 105 cases (98 PPM cases and 7 MMP cases). At the histopathologic examination of the cases with the parental type PPM, 74 of the placentas (76%) were described as molar; 12 placentas (12%) were diagnosed with hydropic degeneration, and 12 placentas (12%) were described as normal placentas. Histopathologic observations were available for 7 of the placentas from pregnancies with the parental type MMP. None of these cases were classified as hydatidiform moles; in 6 cases, the placentas were described as normal, and in 1 case, hydropic degeneration was diagnosed.

Fetal morphologic findings

By US before termination of the pregnancy, a living fetus was registered in 10% of the PPM cases (11/103) and in 95% of the MMP cases (18/19). Among 29 cases with a living fetus at US, fetal abnormalities were registered in 14 cases. Of these, 3 cases had the parental type PPM, and 11 cases had the type MMP; the difference was not significant ($P = .16$). Autopsy data on the fetuses are shown in Table 2. A wide variety of phenotypes that ranged from near normal to highly abnormal was seen.

MS-hCG

In 7 of 24 cases with the parental type MMP, data on MS-beta hCGs were retrieved (median, 0.17 MoM; range, 0.03—0.33 MoM). Among the 134 cases with the parental type PPM, MS-se-total hCGs were retrieved in 75 cases (median, 13.5 MoM; range, 0.25—1556.9 MoM; Figure).

COMMENT

In Denmark, the health care system is public, and all citizens have a unique Civil Registration System number. Therefore, a systematic retrieval of samples and clinical data was possible. Ascertainment bias was minimized because cases were included consecutively and prospectively both in the DMP and in the DCCR.

The real frequency of triploidy is not known. An estimate of 1-2% of all clinically recognizable pregnancies, however, is accepted widely.[1] Within the study period, there were a total of 606,512 live births in Western Denmark. The rate of induced and spontaneous abortions totals 60% of the birth-rate,[20,21] giving an estimate of approximately 1.5 million recognizable pregnancies. We observed 183 triploid pregnancies, which equals 0.1% of all estimated clinically recognizable pregnancies and suggests that the frequency of triploid pregnancies previously has been overestimated. However, some triploid pregnancies are most likely missing in our cohort. In our study, no pregnancy terminated earlier than GA_{LMP} 10 weeks was included. In addition, it is likely that not all triploid pregnancies that were terminated at higher gestational ages were included. Therefore, our data can by no means be taken as representative for "all triploid pregnancies." However, because the triploid pregnancies diagnosed in Western Denmark are either registered in the DMP or the DCCR (or both), our observations are representative of triploid pregnancies that have been detected in a clinical setting.

The frequency of pregnancies with the 2 parental types

Reported frequencies of pregnancies with the 2 different parental types have varied pronouncedly (Table 3). This variation most likely is due to differences in ascertainment. In the present study, we have found a striking predominance of cases with the parental type PPM (85%), but it should be pointed out that

TABLE 3
Studies that estimated frequencies of the 2 parental types in triploid pregnancies

Study	Cohort	Wks' gestation, range (median)	Population	Period	No. registered	No. examined	Method	Frequency of parental type: double paternal contribution, %
Szulman et al[7]	Karyotyped spontaneous abortion	6–27 (NA)	Laboratories Centres dÉtudes de Biologie Prénatale Paris, France	1968-1971	NA	92	Ratios between gonosomes	More frequent than double maternal contribution
Jacobs et al[1]	Karyotyped spontaneous abortion	7–31 (14)	One hospital, Honolulu, HI	1978-1982	106	78	Cytogenetic comparison of Q and C band heteromorphisms with those in parents	73
Uchida and Freeman[8]	Karyotyped spontaneous abortion; abortions, liveborn, and stillborn infants suspected of chromosomal abnormality	NA	McMaster University of Health Sciences Center, Hamilton, Canada	1977-1984	105	76	Cytogenetic comparison of Q and C band heteromorphisms with those in parents	62
McFadden et al[4]	Karyotyped specimens with intact fetus	10–29 (NA)	Embryo Pathology Laboratory and Autopsy Service at British Colombia Children's' Hospital, Canada	1987-1990	110	8	Comparison of DNA markers with those in both parents	25
Miny et al[6]	Chorionic villus samples; karyotyped because of maternal age or abnormal sonography	8–33 (18)	Münster Chorionic Villus Sampling and Amniocentesis Program, Germany	NA	NA	17	Comparison of DNA markers with those in both parents	29
Baumer et al[2]	Karyotyped 22 fetuses; karyotyped 3 liveborn probands	8–36 (20)	NA	NA	NA	25	Comparison of DNA markers with those in both parents	20

Joergensen. Classification of triploid pregnancies. Am J Obstet Gynecol 2014.

(continued)

our estimate can be affected also by ascertainment bias, despite the fact that we have tried to ensure that we included all cases of triploidy from a defined area of Denmark by ascertaining from both the DMP and the DCCR.

Karyotypes

In pregnancies with the parental type MMP, we found no significant difference between the frequencies of the 2 possible karyotypes 69,XXX and 69,XXY ($P = .36$). Similar results have been found by others.[1,2,4]

In triploid pregnancies with the parental type PPM, it generally is believed that the cause behind these is dispermy.[9] If this is true, it would be expected that the frequency of pregnancies with the various karyotypes would be 25% 69,XYY, 50% 69,XXY, and 25% 69,XXX. We found the karyotype 69,XYY in 6.1% of the pregnancies with the parental type PPM. This frequency is significantly higher than the frequency of 1% found by others[1,11,13,22] and at the same time very far from the expected frequency of 25%. Interestingly, the frequency in our study is similar to the frequency that has been observed in preimplanted human triploid embryos.[23] The difference in the frequencies of pregnancies with the karyotype 69,XYY observed by us and others may be explained by a difference in the frequencies of hydatidiform moles in the cohorts because all of the 7 cases with the karyotype 69,XYY in our study were classified as hydatidiform moles. Therefore, one could speculate that preimplantation growth and the growth of a hydatidiform mole require the function of fewer genes compared with the growth of a more differentiated conceptus that is less sensitive to the suggested selection against pregnancies with a deficiency of X chromosomal genes relative to autosomal genes in triploid pregnancies.[2]

However, disregarding the assumed selection against pregnancies with the karyotype 69,XYY, dispermy should still result in twice as many pregnancies with the karyotype 69,XXY as pregnancies with the karyotype 69,XXX. In our study, pregnancies with these 2 karyotypes had similar frequencies, which raises the

TABLE 3
Studies that estimated frequencies of the 2 parental types in triploid pregnancies *(continued)*

Study	Cohort	Wks' gestation, range (median)	Population	Period	No. registered	No. examined	Method	Frequency of parental type: double paternal contribution, %
Zaragoza et al[9]	Karyotyped spontaneous abortion; abortions ascertained because of pathologic observations	5–19 (12)	University Hospitals, Cleveland, OH, and Pittsburg, PA	NA	91	87	Comparison of DNA-markers with those in 1 or both parents	69
McFadden and Langlois[5]	Karyotyped spontaneous abortion; abortions ascertained because of pathologic observations	NA–30 (NA)	Embryo pathology Laboratory and Autopsy Service at British Colombia Children's Hospital, Canada; 1 case from another institution in the United States	NA	41	38	Comparison of DNA markers with those in 1 or both parents	37
Daniel et al[3]	Karyotyped spontaneous abortion; abortions ascertained because of pathologic observations; 1 dysmorphic child	NA	NA	NA	19	18	Phenotype or comparison of DNA-markers with those in 1 or both parents or chromosome 15 methylation test	61
Present study	Triploid pregnancies registered in the Danish Cytogenetic Central Register[a] or the Danish Mole Project[b]	10–30 (16)	Western Denmark	April 1986–April 2010	183	158	Methylation sensitive multiplex ligation–dependent probe	85

In the genetic laboratory, the placental tissue was inspected, and only samples with at least 10 vesicular chorionic villi with diameters of $\geq$1 mm were included. Ploidy is determined by karyotyping of uncultured and/or cultured cells and/or by measurement of the nuclear DNA contents by flow cytometry of unfixed nuclei, with 2 external controls.

NA, not available.

[a] Holds data on all karyotypes made for clinical purposes in Denmark since 1960; [b] Holds data on and samples of fresh placental tissue from conceptuses that gynecologists suspected to be hydatidiform moles.

Joergensen. Classification of triploid pregnancies. Am J Obstet Gynecol 2014.

question whether dispermy is the only mechanism that causes PPM triploidy.

Placenta morphologic findings

Combining the data on triploid pregnancies of both parental types, molar appearance was found on US in 75 of 103 cases (73%). Our results contrast to the observation of molar appearance on US in 30% in triploid pregnancies, which was reported by Jauniaux et al.[10] Our cohort consisted of all triploid pregnancies that were detected in a clinical setting in a well-defined geographic region. However, because the Departments of Gynecology participated in a research project on hydatidiform moles, the gynecologists may very well have been focused especially on forwarding samples with a molar morphologic finding for genetic analyses. This may have resulted in an overestimation of the proportion of molar placentas from triploid pregnancies in our study. In the study by Jauniaux et al,[10] patients who were referred with placental abnormalities were excluded. This should result in an underestimation of the proportion of placentas with molar appearance. Thus, it seems plausible that the proportion of triploid conceptuses with molar appearance on US is somewhere between 30% and 59%.

Suspicion of a molar pregnancy based on US was raised in 75 of 103 PPM cases and in none of 22 MMP cases. Thus, in our cohort, the specificity and sensitivity for the detection of diandric triploids was 100% and 70%, respectively. All cases that fulfilled the DMP inclusion criteria had the parental type PPM, which indicates that this selection is highly specific for the parental type PPM.

At histopathologic examination of the 105 placentas, 74 were diagnosed as molar; they all had the parental type PPM. Among the 98 cases with the parental type PPM, 76% were molar; 12% were diagnosed with hydropic degeneration, and 12% were normal placentas. This is in agreement with the results from histopathologic examinations of spontaneous abortions with the parental type PPM reported by Zaragoza et al.[9] In our cohort of triploid

pregnancies, a histopathologic diagnosis of molar pregnancy would have had a specificity of 100% and a sensitivity of 76% for the parental type PPM.

The correlation between morphologic findings and parental type may have been influenced by the high frequency of PPM cases in our cohort. However, it seems safe to conclude that the specificity of suspicion of molar pregnancy by any of the morphologic methods for the prediction of diandric triploidy is high.

In contrast to others, we did not find a correlation between small placentas and the parental type MMP.[4] However, we had information only on histopathologic morphologic findings of the placenta in 7 cases with the parental type MMP; hence, our results show only that normal placentas can be observed in triploid pregnancies with the parental type MMP.

Fetal morphologic findings

The frequency of fetal abnormalities detected by US in triploid pregnancies with a living fetus has been stated to be 93%,[24] 66%,[10] or 86%.[23] In our study, fetal abnormalities at the initial US were registered in 48% (95% confidence interval, 29–69%) of the triploid pregnancies with a living fetus. The mean gestational age is available for 2 of the earlier studies: 11 and 20 weeks, respectively.[10,24] The mean GA_{LMP} in our study was 15 weeks. Thus, it is not likely that differences in gestational age explain the differences in the observed frequencies of triploid pregnancies with fetal abnormalities that are detected by US. However, ascertainment bias cannot be excluded as a cause of the discrepancies.

A variety of fetal phenotypes that ranged from nearly normal to highly abnormal were seen. No malformations that are pathognomonic for triploid fetuses were seen, which correlates well with the findings in previous studies.[3,4,22]

Two distinct phenotypes have been proposed to be associated with triploidy after the gestational age 10 + 0 weeks: type 1 (with the parental type PPM) consists of relatively well-grown fetuses with proportionately sized body parts, and the associated placentas are abnormally large with the phenotype of partial hydatidiform mole; and type 2 (with the parental type MMP) consists of growth-retarded fetuses with pronounced wasting of the body and therefore relative macrocephaly; the associated placentas are noncystic and abnormally small.[4,14]

A substantial fraction of our cases, which all had GA_{LMP} above 10 + 0 weeks, did not comply with this categorization. In 6 of 8 PPM cases (75%) we found fetuses with IUGR; in 3 cases (38%), the placenta was normal. In 2 of 7 MMP cases (29%), no IUGR was found; in 4 of the 7 cases (57%), the placenta was normal.

In our study, a fetus was classified as subject to IUGR if the gestational age according to ultrasound biometric parameters was <7 days shorter than that calculated from the last menstrual period. Others have defined IUGR using development stage of the fetus that was seen in autopsy.[4,14] Therefore, comparison of IUGRs must be made with caution. However, the combination of results from placental examination and IUGR data prompts us to suggest that the association of 2 distinct phenotypes with the 2 parental types is not entirely consistent.

MS-hCG

Most studies have reported high MS-total hCG in cases with the parental type PPM.[4,11,25,26] Although we observed high values in many PPM cases, we also observed some PPM cases with low MS-total hCG values, which corroborates the observations in previous case reports.[12,24] Like others,[11,18,25,26] we found a low MS-beta hCG in all the triploid pregnancies with the parental type MMP. Thus, the use of a high MS-beta hCG value in a triploid pregnancy as a predictor of the parental type PPM has a high specificity, but a lower sensitivity.

By the correlation of triploid pregnancy and either suspicion of hydatidiform mole at US, at macroscopic inspection of the evacuated tissue, or at histologic examination and a high level MS-beta hCG, one can predict the parental type PPM with a very high specificity. In contrast, the sensitivity of these observations as predictors of this parental type is <100%. ■

ACKNOWLEDGMENTS

The authors are grateful for the assistance from Professor Steen Kolvraa, DMSc, MedDr, the Department of Clinical Genetics, Vejle Hospital, and Institute of Regional Health Research, University of Southern Denmark for discussions and assistance in constructing the manuscript, and from Associate Professor Niels Tørring, PhD, at the Department of Clinical Biochemistry, Aarhus University Hospital-Skejby, for providing algorithms for calculations of MoM values. We are also grateful for the help supplied from the Departments of Gynecology and Obstetrics in Jutland when old medical records had to be retrieved. Finally, we would like to express our gratitude to the Departments of Clinical Genetics in Aarhus and Vejle for their help in retrieving triploid samples.

REFERENCES

1. Jacobs PA, Szulman AE, Funkhouser J, Matsuura JS, Wilson CC. Human triploidy: relationship between parental origin of the additional haploid complement and development of partial hydatidiform mole. Ann Hum Genet 1982;46:223-31.
2. Baumer A, Balmer D, Binkert F, Schinzel A. Parental origin and mechanisms of formation of triploidy: a study of 25 cases. Eur J Hum Gene 2000;8:911-7.
3. Daniel A, Wu Z, Bennetts B, et al. Karyotype, phenotype and parental origin in 19 cases of triploidy. Prenat Diagn 2001;21:1034-48.
4. McFadden DE, Kwong LC, Yam IY, Langlois S. Parental origin of triploidy in human fetuses: evidence for genomic imprinting. Hum Genet 1993;92:465-9.
5. McFadden DE, Langlois S. Parental and meiotic origin of triploidy in the embryonic and fetal periods. Clin Genet 2000;58:192-200.
6. Miny P, Koppers B, Dworniczak B, et al. Parental origin of the extra haploid chromosome set in triploidies diagnosed prenatally. Am J Med Genet 1995;57:102-6.
7. Szulman AE, Philippe E, Boue JG, Boue A. Human triploidy: association with partial hydatidiform moles and nonmolar conceptuses. Hum Pathol 1981;12:1016-21.
8. Uchida IA, Freeman VC. Triploidy and chromosomes. Am J Obstet Gynecol 1985;151:65-9.
9. Zaragoza MV, Surti U, Redline RW, Millie E, Chakravarti A, Hassold TJ. Parental origin and phenotype of triploidy in spontaneous abortions: predominance of diandry and association with the partial hydatidiform mole. Am J Hum Genet 2000;66:1807-20.
10. Jauniaux E, Brown R, Rodeck C, Nicolaides KH. Prenatal diagnosis of triploidy during the second trimester of pregnancy. Obstet Gynecol 1996;88:983-9.

11. Huang T, Alberman E, Wald N, Summers AM. Triploidy identified through second-trimester serum screening. Prenat Diagn 2005;25:229-33.
12. Oyer CE, Canick JA. Maternal serum hCG levels in triploidy: variability and need to consider molar tissue. Prenat Diagn 1992;12:627-9.
13. McFadden DE, Hulait G, Lockitch G, Langlois S. Maternal serum screening in triploidy. Prenat Diagn 2002;22:1113-4.
14. McFadden DE, Kalousek DK. Two different phenotypes of fetuses with chromosomal triploidy: correlation with parental origin of the extra haploid set. Am J Med Genet 1991;38:535-8.
15. Sunde L, Mogensen B, Olsen S, Nielsen V, Christensen IJ, Bolund L. Flow cytometric DNA analyses of 105 fresh hydatidiform moles, with correlations to prognosis. Anal Cell Pathol 1996;12:99-114.
16. Joergensen MW, Rasmussen AA, Niemann I, et al. Methylation specific multiplex ligation-dependent probe amplification: utility for prenatal diagnosis of parental origin in human triploidy. Prenat Diagn 2013;33:1131-6.
17. Baskett TF, Nagele F. Naegele's rule: a reappraisal. BJOG 2000;107:1433-5.
18. Spencer K. First trimester maternal serum screening for Down's syndrome: an evaluation of the DPC Immulite 2000 free beta-hCG and pregnancy-associated plasma protein-A assays. Ann Clin Biochem 2005;42:30-40.
19. Siemens IMMULITE HCG recommended median settings for PRISCA in Europe (HCG Trisomy 21 Application Sheet (ZI003-6, 2009-12-16)). Munich, Bavaria: Siemens.
20. Statistikbanken. Available at: http://www.statistikbanken.dk/statbank5a/default.asp?w=1680. Accessed Sept. 25, 2013.
21. Information om gentagne spontane aborter. Available at: http://www.rigshospitalet.dk/menu/AFDELINGER/Juliane+Marie+Centret/Klinikker/Fertilitetsklinikken/Enheden+for+gentagne+aborter/Generel_information_til_patienter.htm. Accessed Sept. 25, 2013.
22. Doshi N, Surti U, Szulman AE. Morphologic anomalies in triploid liveborn fetuses. Hum Pathol 1983;14:716-23.
23. McWeeney DT, Munne S, Miller RC, et al. Pregnancy complicated by triploidy: a comparison of the three karyotypes. Am J Perinatol 2009;26:641-5.
24. Jauniaux E, Brown R, Snijders RJ, Noble P, Nicolaides KH. Early prenatal diagnosis of triploidy. Am J Obstet Gynecol 1997;176:550-4.
25. Eiben B, Hammans W, Goebel R. Triploidy, imprinting, and hCG levels in maternal serum screening. Prenat Diagn 1996;16:377-8.
26. Jauniaux E. Partial moles: from postnatal to prenatal diagnosis. Placenta 1999;20:379-88.
27. Fukunaga M, Katabuchi H, Nagasaka T, Mikami Y, Minamiguchi S, Lage JM. Interobserver and intraobserver variability in the diagnosis of hydatidiform mole. Am J Surg Pathol 2005;29:942-7.
28. Howat AJ, Beck S, Fox H, et al. Can histopathologists reliably diagnose molar pregnancy? J Clin Pathol 1993;46:599-602.
29. Javey H, Borazjani G, Behmard S, Langley FA. Discrepancies in the histological diagnosis of hydatidiform mole. BJOG 1979;86:480-3.

Appendix

SUPPLEMENTARY TABLE

Parental origin, karyotype, and phenotype of triploid pregnancies

Identification no.[a]	Parenteral type	Karyotype	GA_{LMP} at first US, wk + d	GA_{CRL} first US, wk + d	⊿ GA_{LMP} vs GA_{CRL}, d	First US	Supplementary US	Histopathologic diagnosis of placenta[b]	GA_{LMP} at termination, wk + d	Maternal age, y	GA_{LMP} at hCG	hCG MoM[c]
1[a]	MMP	69,XXY	12 + 3	12	−3	Asymmetric IUGR, holoprosencephallic fetus with vague facial profile		Hydropic degeneration	13	33		
2[a]	MMP	69,XXY	12 + 4	11 + 2	−9	Possible asymmetrical IUGR			15	39	9 + 6	0.33[c]
3[a]	MMP	69,XXY		21 + 4		IUGR? Possible transposition of the great arteries or double outlet right ventricle, misalignment of wrists, and overlapping fingers			22	29		
4[a]	MMP	69,XXY	NA	NA	NA	NA	15 d after first US: no FHA, GA 10 + 5		10 + 5	41		
5[a]	MMP	69,XXX	12 + 5	10 + 1	−18	Early IUGR			13 + 1	28		
6[a]	MMP	69,XXY	13 + 1	12 + 1	−7	No abnormalities detected			13 + 6	35		
7[a]	MMP	69,XXX	11 + 5	11 + 4	−1	No abnormalities detected	12 d after first US: no abnormalities detected; 2 d after: asymmetrical IUGR		14 + 2	27	10 + 1	0.3[c]
8[a]	MMP	69,XXX	15 + 2	13 + 4	−12	Small placenta, oligohydramnios, asymmetrical IUGR			15 + 4	27	12 + 6	0.13[c]
9[a]	MMP	69,XXX	14 + 2			Possible IUGR	5 d after first US: IUGR; 1 wk after: no FHA		15 + 5	37	14 + 4	0.03[c]
10[a]	MMP	69,XXX	NA	10 + 6	NA	Thin small placenta	1 mo after first US: asymmetric IUGR, hydrocephalic fetus with lemon sign, echogenic cardiac foci ("golf balls") and overlapping fingers		16 + 1	39		

(continued)

SUPPLEMENTARY TABLE

Parental origin, karyotype, and phenotype of triploid pregnancies *(continued)*

Identification no.[a]	Parenteral type	Karyotype	GA_{LMP} at first US, wk + d	GA_{CRL} first US, wk + d	⊿ GA_{LMP} vs GA_{CRL}, d	First US	Supplementary US	Histopathologic diagnosis of placenta[b]	GA_{LMP} at termination, wk + d	Maternal age, y	GA_{LMP} at hCG	hCG MoM[c]
11[a]	MMP	69,XXY	12 + 4	11 + 1	−10	No abnormalities detected	11 d after first US: asymmetric IUGR; 13 d after: no FHA		16 + 3	30	12 + 5	0.17[c]
12[a]	MMP	69,XXX	11 + 6	10 + 4	−9	Oligohydramnios	8 d after first US: oligohydramnios; asymmetric IUGR		16 + 6	32	9 + 6	0.48[c]
13[a]	MMP	69,XXX	13 + 4	12 + 1	−10	Possible IUGR	16 d after first US: no abnormalities detected; 10 d after: no FHA		17 + 3	30		
14[a]	MMP	69,XXX		10		NA	2 mo after first US: large placenta, single umbilical artery, negative blood flow in diastole; oligohydramnios, asymmetrical IUGR		18 + 4	23		
15[a]	MMP	69,XXY	16 + 5	14 + 2	−17	No abnormalities detected	2 mo after first US: IUGR; 10 d after: IUGR and slow heart rate		26 + 3	29		
16[a]	MMP	69,XXY				No abnormalities detected				35		
17[a]	MMP									NA		
18[a]	MMP	69,XXY	12	10 + 4	−10	Possible oligohydramnic	14 d after first US: oligohydramnios, asymmetric IUGR, possible hydrocephalic	Normal	15 + 2	31		
19[a]	MMP	69,XXX	15 + 2	14	−9	Asymmetrical IUGR		Normal	15 + 3	29		
20[a]	MMP	69,XXX	13 + 1	11 + 6	−9	No abnormalities detected	1 mo after first US: oligohydramnios, IUGR, echogenic cardiac foci ("golf balls")	Normal	15 + 3	32	13 + 1	0.17[c]
21[a]	MMP	69,XXX	20 + 1	17 + 5	−17	Oligohydramnios, IUGR, hydrocephalic fetus		Normal	20 + 5	27		

(continued)

SUPPLEMENTARY TABLE

Parental origin, karyotype, and phenotype of triploid pregnancies *(continued)*

Identification no.[a]	Parenteral type	Karyotype	GA_{LMP} at first US, wk + d	GA_{CRL} first US, wk + d	Δ GA_{LMP} vs GA_{CRL}, d	First US	Supplementary US	Histopathologic diagnosis of placenta[b]	GA_{LMP} at termination, wk + d	Maternal age, y	GA_{LMP} at hCG	hCG MoM[c]
22[a]	MMP	69,XXY	16 + 6	14	−20	Oligohydramnios	2 wks after first US: oligohydramnios; 1 mo after second US: oligohydramnios, asymmetric IUGR	Normal	23 + 6	26		
23[a]	MMP	69,XXX	11 + 1	10 + 6	−2	No abnormalities detected	4 mos after first US: oligohydramnios, asymmetric IUGR	Normal	30 + 3	30		
24[a]	MMP	Double paternal contribution										
25	PPM		15			Fetus with no FHA		Complete mole	15	40	15	4.02
26	PPM		17 + 6			Placenta with molar characteristics; no fetus		Complete mole	18	22	18	11.35
27	PPM		12 + 5			Spontaneous abortion		Complete mole	12 + 5	20	12 + 5	
28	PPM	69,XXX	14			Placenta with molar characteristics; no fetus		Complete mole	14 + 1	33	14 + 1	0.25
29	PPM	69,XXX	14 + 4			Placenta with molar characteristics; no fetus		Complete mole	14 + 5	26	14 + 5	38.55
30	PPM	NA	15			Placenta with molar characteristics; no fetus		Complete mole	15 + 1	35	15 + 1	18.27
31	PPM	69,XXY	15			Spontaneous abortion		Complete mole	15 + 1	25	15 + 1	13.21
32	PPM	69,XYY	16 + 4			Spontaneous abortion		Complete mole	16 + 5	24	16 + 5	
33	PPM	69,XXY	17			Placenta with molar characteristics; fetus with no FHA; GA, 17 wks		Complete mole	17 + 1	30	17 + 1	15.54
34	PPM	69,XXX	18			Placenta with molar characteristics; no fetus		Complete mole	18 + 1	24	18 + 1	
35	PPM		17 + 6			Placenta with molar characteristics; no fetus		Complete mole	18 + 3	26	18 + 3	9.74
36	PPM	69,XXY	18 + 4			Spontaneous abortion		Complete mole	18 + 4	26	18 + 4	2.09
37	PPM	69,XXY	19 + 1			Placenta with molar characteristics; no fetus		Complete mole	19 + 2	38	19 + 2	12.2

(continued)

SUPPLEMENTARY TABLE

Parental origin, karyotype, and phenotype of triploid pregnancies *(continued)*

Identification no.[a]	Parenteral type	Karyotype	GA_{LMP} at first US, wk + d	GA_{CRL} first US, wk + d	Δ GA_{LMP} vs GA_{CRL}, d	First US	Supplementary US	Histopathologic diagnosis of placenta[b]	GA_{LMP} at termination, wk + d	Maternal age, y	GA_{LMP} at hCG	hCG MoM[c]
38	PPM	69,XXX	17 + 6			Placenta with molar characteristics; no fetus		Pregnancy product, not specified	18	28	18	15.45
39	PPM	69,XXY	12			Placenta with molar characteristics; no fetus		Pregnancy product, not specified	12 + 1	23	12 + 1	8.79
40	PPM	69,XXY	13 + 5			Fetus with no FHA; GA, 14 wks		Pregnancy product, not specified	13 + 6	30	13 + 6	54.2
41	PPM	69,XXY	13 + 6			Placenta with molar characteristics; no fetus		Pregnancy product, not specified	13 + 6	29	13 + 6	2.94
42	PPM	69,XXX	15 + 1			Placenta with molar characteristics; fetus with no FHA; GA, 9 wks		Pregnancy product, not specified	15 + 1	31	15 + 1	
43	PPM	69,XXY	15 + 1			Placenta with molar characteristics; fetus with no FHA; GA, 8 wks + 5 d		Pregnancy product, not specified	15 + 2	24	15 + 2	
44	PPM	69,XXX	15 + 2			Placenta with molar characteristics; fetus (not specified)		Pregnancy product, not specified	15 + 3	26	15 + 3	
45	PPM		16 + 2			Spontaneous abortion		Pregnancy product, not specified	16 + 2	27	16 + 2	1.4
46	PPM	69,XXY	16 + 2			Placenta with molar characteristics; fetus with no FHA		Pregnancy product, not specified	16 + 3	31	16 + 3	14.77
47	PPM	69,XXY	12			Placenta with molar characteristics; no fetus		Pregnancy product, not specified	16 + 4	19	16 + 4	104.28
48	PPM	69,XYY	15			Placenta with molar characteristics; no fetus		Hydropic degeneration	15	29	15	15.81
49	PPM	69,XXY	16 + 4			Placenta with molar characteristics; fetus with no FHA		Hydropic degeneration	17	24	17	12.8

(continued)

SUPPLEMENTARY TABLE

Parental origin, karyotype, and phenotype of triploid pregnancies *(continued)*

Identification no.[a]	Parenteral type	Karyotype	GA_{LMP} at first US, wk + d	GA_{CRL} first US, wk + d	ΔGA_{LMP} vs GA_{CRL}, d	First US	Supplementary US	Histopathologic diagnosis of placenta[b]	GA_{LMP} at termination, wk + d	Maternal age, y	GA_{LMP} at hCG	hCG MoM[c]
50	PPM		15					Hydropic degeneration	15 + 1	28	15 + 1	
51	PPM	69,XXX	15 + 3			Spontaneous abortion		Hydropic degeneration	15 + 3	30	15 + 3	
52	PPM	69,XXX	16 + 2			Placenta with molar characteristics; fetus with no FHA		Hydropic degeneration	16 + 3	25	16 + 3	
53	PPM	69,XYY	17			Placenta with molar characteristics; no fetus		Hydropic degeneration	17 + 1	19	17 + 1	34.41
54	PPM	69,XXY	17 + 2			Placenta with molar characteristics; fetus with no FHA		Hydropic degeneration	17 + 2	19	17 + 2	10.94
55	PPM	69,XXX	17 + 3			Fetus with no FHA		Hydropic degeneration	17 + 4	25	17 + 4	
56	PPM	69,XXY	18			Placenta with molar characteristics; no fetus		Hydropic degeneration	18 + 1	23	18 + 1	20.07
57	PPM	69,XXX	18 + 2			Placenta with molar characteristics; no fetus		Hydropic degeneration	18 + 3	23	18 + 3	21.09
58	PPM		18 + 3			Placenta with molar characteristics; fetus with no FHA; GA, 11 wk		Hydropic degeneration	18 + 4	27	18 + 4	
59	PPM	69,XXY	18 + 3			Placenta with molar characteristics, no FHA, asymmetric IUGR with sparring of head		Hydropic degeneration	18 + 4	25	18 + 4	1.15
60	PPM	69,XXY	12 + 4			Fetus with no FHA; GA, 12 wk		Molar not specified	12 + 4	31	12 + 4	8.03
61	PPM	69,XXX	16 + 5			Placenta with molar characteristics; no fetus		Molar not specified	16 + 6	31	16 + 6	159.75
62	PPM	69,XXY	18 + 3			Placenta with molar characteristics; no fetus		Molar not specified	18 + 5	37	18 + 5	11.4
63[a]	PPM	69,XXX	14 + 5	12 + 6	−13	No abnormalities detected	18 d after first US: GA, 15 + 3 wk; IUGR; possible heart malformation		17 + 5	39		

(continued)

SUPPLEMENTARY TABLE

Parental origin, karyotype, and phenotype of triploid pregnancies *(continued)*

Identification no.[a]	Parenteral type	Karyotype	GA_{LMP} at first US, wk + d	GA_{CRL} first US, wk + d	Δ GA_{LMP} vs GA_{CRL}, d	First US	Supplementary US	Histopathologic diagnosis of placenta[b]	GA_{LMP} at termination, wk + d	Maternal age, y	GA_{LMP} at hCG	hCG MoM[c]
64[a]	PPM	69,XXY				Cystic hygroma				30		
65[a]	PPM	69,XXX		11 + 4		No abnormalities detected	19 d after first US: placenta with molar characteristics; fetus with possible heart defects			28		
66[a]	PPM	69,XXY				NT 3.7 mm				27		
67[a]	PPM	69,XXY	NA			Omphalocele				33		
68[a]	PPM									NA		
69[a]	PPM	69,XXX	12 + 2	12	−2	NT, 6 mm				35		
70[a]	PPM	69,XXX	9	9	0	No abnormalities detected	1 wk after first US: no abnormalities detected; 2 wk after second US: placenta with molar characteristics; no FHA	Normal	12 + 2	39		
71	PPM	69,XXY	16 + 5			Placenta with molar characteristics; fetus without abnormalities	7 d after first US: GA, 15 + 3 wk; placenta with molar characteristics	Normal	18 + 3	32	18 + 6	1339.5
72	PPM	69,XXY	12			Fetus with no FHA		Partial mole	12	34	12	47.54
73	PPM	69,XXY	14			Placenta with molar characteristics; no fetus		Partial mole	12	26	12	5.45
74	PPM	69,XXY	12 + 6			Fetus with no FHA		Partial mole	13	31	13	8.8
75	PPM	69,XXX	16 + 5			Fetus with no FHA		Partial mole	17	38	17	10.43
76[a]	PPM	69,XXY	16 + 4			Fetus with no FHA		Partial mole	19	36	16 + 4	
77	PPM	69,XXY	18 + 6			Placenta with molar characteristics; fetus (not specified)		Partial mole	19	30	19	
78	PPM	69,XXX	19			Placenta with molar characteristics; no fetus		Partial mole	19	24	19	7.09
79	PPM	69,XXY	19 + 6			Placenta with molar characteristics; fetus with no FHA		Partial mole	20	25	20	3.85

(continued)

SUPPLEMENTARY TABLE

Parental origin, karyotype, and phenotype of triploid pregnancies *(continued)*

Identification no.[a]	Parenteral type	Karyotype	GA_{LMP} at first US, wk + d	GA_{CRL} first US, wk + d	Δ GA_{LMP} vs GA_{CRL}, d	First US	Supplementary US	Histopathologic diagnosis of placenta[b]	GA_{LMP} at termination, wk + d	Maternal age, y	GA_{LMP} at hCG	hCG MoM[c]
80	PPM	69,XXY	20 + 6			Placenta with molar characteristics; no fetus		Partial mole	21	26	21	0.54
81	PPM	69,XXX	21 + 6			Spontaneous abortion		Partial mole	22	24	22	
82	PPM	69,XXY	11 + 3			Placenta with molar characteristics; fetus with no FHA		Partial mole	12 + 5	30	12 + 5	17.87
83	PPM	69,XXX	12 + 5			Placenta with molar characteristics; no fetus		Partial mole	12 + 6	32	12 + 6	
84	PPM	69,XXX	12 + 6			Placenta with molar characteristics; no fetus		Partial mole	12 + 6	30	12 + 6	23.29
85	PPM	69,XXY	13 + 3			Placenta with molar characteristics; no fetus		Partial mole	13 + 4	19	13 + 4	13.72
86[a]	PPM	69,XXX	13			Placenta with molar characteristics; fetus NT, 6 mm		Partial mole	13 + 6	26	12 + 6	382.22
87	PPM	69,XXX	14			Placenta with molar characteristics; fetus with no FHA		Partial mole	14 + 1	29	14 + 1	
88	PPM	69,XXX	14 + 2			Placenta with molar characteristics; fetus with no FHA		Partial mole	14 + 3	28	14 + 3	53.4
89	PPM	69,XXY	14 + 4			Fetus with no FHA; GA, 8 wk		Partial mole	14 + 5	23	14 + 5	54.65
90	PPM	69,XXY	15 + 1			Placenta with molar characteristics; no fetus		Partial mole	15 + 2	27	15 + 2	5.06
91	PPM	69,XXY	15 + 2			Fetus with no FHA; GA, 15 wk		Partial mole	15 + 3	29	15 + 3	8.37
92	PPM	69,XXY	15 + 3			Placenta with molar characteristics; fetus with no FHA		Partial mole	15 + 3	35	15 + 3	8.18
93	PPM	69,XXY	15 + 5			Spontaneous abortion		Partial mole	15 + 5	31	15 + 5	1.41
94	PPM	69,XXX	16			Fetus with no FHA; GA, 11 wk		Partial mole	16 + 1	33	16 + 1	

(continued)

SUPPLEMENTARY TABLE

Parental origin, karyotype, and phenotype of triploid pregnancies *(continued)*

Identification no.[a]	Parenteral type	Karyotype	GA_{LMP} at first US, wk + d	GA_{CRL} first US, wk + d	Δ GA_{LMP} vs GA_{CRL}, d	First US	Supplementary US	Histopathologic diagnosis of placenta[b]	GA_{LMP} at termination, wk + d	Maternal age, y	GA_{LMP} at hCG	hCG MoM[c]
95	PPM		16			Placenta with molar characteristics; no fetus		Partial mole	16 + 1	30	16 + 1	
96	PPM	69,XXY	15 + 4			Placenta with molar characteristics; fetus with FHA; GA, 13 wk		Partial mole	16 + 2	25	16 + 2	955.81
97	PPM		16 + 2			Placenta with molar characteristics; no fetus		Partial mole	16 + 2	19	16 + 2	
98	PPM	69,XXY	16 + 2			Spontaneous abortion		Partial mole	16 + 3	34	16 + 3	27.99
99	PPM	69,XXY	16 + 4			Placenta with molar characteristics; fetus with no FHA		Partial mole	16 + 6	27	16 + 6	
100	PPM	69,XXY	16 + 5			Placenta with molar characteristics; fetus with no FHA		Partial mole	16 + 6	30	16 + 6	0.31
101	PPM	69,XXY	16 + 5			Placenta with molar characteristics; fetus with no FHA		Partial mole	16 + 6	32	16 + 6	16.57
102	PPM	69,XXY	17			Placenta with molar characteristics; no fetus		Partial mole	17 + 1	29	17 + 1	14.6
103	PPM	69,XXX	17 + 1			Fetus with no FHA		Partial mole	17 + 2	29	17 + 2	
104	PPM	69,XXX	17			Placenta with molar characteristics; no fetus		Partial mole	17 + 2	30	17 + 2	26.78
105	PPM	69,XXY	17 + 2			Placenta with molar characteristics; fetus with no FHA; GA, 12-13 wk		Partial mole	17 + 3	26	17 + 3	19.45
106	PPM		14 + 6			Spontaneous abortion		Partial mole	17 + 4	26	17 + 4	1.47
107	PPM	69,XXY	17 + 4			NA		Partial mole	17 + 5	28	17 + 5	
108	PPM	69,XXX	17 + 5			Placenta with molar characteristics; fetus with no FHA		Partial mole	17 + 5	23	17 + 5	85.72
109	PPM	69,XXX	17 + 3			Placenta with molar characteristics; no fetus		Partial mole	17 + 5	23	17 + 5	1556.9

(continued)

SUPPLEMENTARY TABLE

Parental origin, karyotype, and phenotype of triploid pregnancies *(continued)*

Identification no.[a]	Parenteral type	Karyotype	GA_{LMP} at first US, wk + d	GA_{CRL} first US, wk + d	Δ GA_{LMP} vs GA_{CRL}, d	First US	Supplementary US	Histopathologic diagnosis of placenta[b]	GA_{LMP} at termination, wk + d	Maternal age, y	GA_{LMP} at hCG	hCG MoM[c]
110	PPM	69,XXX	18			Placenta with molar characteristics; no fetus		Partial mole	18 + 1	25	18 + 1	197.85
111	PPM	69,XXX	11 + 6			Fetus with no FHA		Partial mole	18 + 2	19	18 + 2	4.76
112	PPM	69,XXX	18 + 2			Fetus with no FHA; GA, 11 wk		Partial mole	18 + 2	30	18 + 2	35.34
113	PPM		18 + 1			Placenta with molar characteristics; no fetus		Partial mole	18 + 2	28	18 + 2	22.46
114	PPM	69,XYY	18 + 2			Placenta with molar characteristics; no fetus		Partial mole	18 + 3	27	18 + 3	22.72
115	PPM	69,XXY	18 + 5			Placenta with molar characteristics; fetus with no FHA		Partial mole	18 + 5	28	18 + 5	3.39
116	PPM		18 + 5			Spontaneous abortion		Partial mole	18 + 6	26	18 + 6	1.43
117	PPM	69,XXX	19 + 1			Fetus with no FHA; GA, 9 wk		Partial mole	19 + 2	25	19 + 2	2.16
118	PPM	69,XXX	19			Placenta with molar characteristics; no fetus		Partial mole	19 + 3	36	19 + 3	80.92
119	PPM		19 + 3			Spontaneous abortion		Partial mole	19 + 3	33	19 + 3	12.2
120	PPM	69,XXX	20			Placenta with molar characteristics; fetus (not specified)		Partial mole	20 + 1	24	20 + 1	131.02
121	PPM	69,XXY	20 + 1			Fetus with no FHA		Partial mole	20 + 2	25	20 + 2	7.45
122	PPM	69,XXX	23 + 5			Placenta with molar characteristics; fetus with no FHA; GA, 14 wk		Partial mole	24 + 5	29	24 + 5	3.52
123[a]	PPM	69,XXY	26 + 2			Supposedly normal 12-week scan (second hand information, direct confirmation not possible)		Partial mole	26 + 2	31		
124[a]	PPM	69,XXY	13 + 3	12 + 2	−8	No abnormalities detected		Partial mole		27		
125	PPM	69,XXX	12			Placenta with molar characteristics; no fetus		Partial mole		31		

(continued)

SUPPLEMENTARY TABLE

Parental origin, karyotype, and phenotype of triploid pregnancies *(continued)*

Identification no.[a]	Parenteral type	Karyotype	GA_{LMP} at first US, wk + d	GA_{CRL} first US, wk + d	Δ GA_{LMP} vs GA_{CRL}, d	First US	Supplementary US	Histopathologic diagnosis of placenta[b]	GA_{LMP} at termination, wk + d	Maternal age, y	GA_{LMP} at hCG	hCG MoM[c]
126	PPM	69,XXX				Placenta with molar characteristics; no fetus		Partial mole		26		
127	PPM	69,XXX	11 + 6			Placenta with molar characteristics; no fetus		Partial mole		25	11 + 6	
128	PPM	69,XXY						Partial mole				
129	PPM	69,XXY						Partial mole				
130	PPM	69,XXY							12 + 6		12 + 6	
131	PPM	69,XXX		12 + 4		Placenta with molar characteristics						
132	PPM	69,XXX		12 + 2		Placenta with molar characteristics						
133	PPM	69,XXX				Placenta with molar characteristics						
134	PPM	69,XXX		12 + 5		Placenta with molar characteristics						
135	PPM	69,XXX		12		Placenta with molar characteristics						
136	PPM	69,XXY		12 + 5		Placenta with molar characteristics						
137	PPM	69,XXY		9 + 4		Placenta with molar characteristics						
138	PPM	69,XYY		9 + 4		Placenta with molar characteristics						
139	PPM	69,XYY		12 + 2		Placenta with molar characteristics						
140	PPM					Placenta with molar characteristics						
141	PPM	69,XXY		12 + 1		Placenta with molar characteristics; no fetus						
142	PPM	69,XXX										
143	PPM	69,XXX										

(continued)

SUPPLEMENTARY TABLE

Parental origin, karyotype, and phenotype of triploid pregnancies *(continued)*

Identification no.[a]	Parenteral type	Karyotype	GA_{LMP} at first US, wk + d	GA_{CRL} first US, wk + d	Δ GA_{LMP} vs GA_{CRL}, d	First US	Supplementary US	Histopathologic diagnosis of placenta[b]	GA_{LMP} at termination, wk + d	Maternal age, y	GA_{LMP} at hCG	hCG MoM[c]
144	PPM	69,XXX										
145	PPM	69,XXX										
146	PPM	69,XXX										
147	PPM	69,XXX		12 + 2								
148	PPM	69,XXY										
149	PPM	69,XXY										
150	PPM	69,XXY										
151	PPM	69,XXY										
152	PPM	69,XXY										
153	PPM	69,XXY										
154	PPM	69,XXY										
155	PPM	69,XYY										
156	PPM											
157	PPM											
158	PPM											

beta hCG, human chorionic gonadotropin; *Δ*, delta; *FHA,* fetal heart activity; *GA*, gestational age; GA_{CRL}, gestational age calculated from crown rump length; GA_{LMP}, gestational age calculated from last menstrual period; *hCG*, human chorionic gonadotropin; *IUGR*, intrauterine growth restriction; *MMP*, double maternal contribution; *MoM*, multiples of the median; *NA*, not available; *PPM*, double paternal contribution; *US*, ultrasound.

[a] From the Danish Cytogenetic Central Registry; [b] Reports on poor histopathologic interobserver and intraobserver reproducibility have been published.[27-29] Because this part of the study was retrospective by many different pathologists over a period of 24 years, we have no detailed information on sampling. Therefore, we have not commented on any potential difference between partial and complete moles because these are especially subject to sampling. Differentiation between the 2 molar diagnoses in this study should be made only with caution; [c] Beta hCG.
