# Supplementary material for: Triploidy—Observations in 154 Diandric Cases
Source: PLoS One. 2015 Nov 12;10(11):e0142545. doi: 10.1371/journal.pone.0142545 (PMC4642992; doi:10.1371/journal.pone.0142545)
Supplement: S2 File — (PDF) [file pone.0142545.s002.pdf]

Available online at www.sciencedirect.com
ScienceDirect
Gynecologic Oncology 104 (2007) 411–415
Gynecologic Oncology
www.elsevier.com/locate/ygyno

The risk of persistent trophoblastic disease after hydatidiform mole classified by morphology and ploidy

Isa Niemann [a,*], Estrid S. Hansen [b], Lone Sunde [a]

[a] Department of Clinical Genetics, Aarhus University Hospital, Bartholin Bygningen, Aarhus Sygehus, 8000 Aarhus C, Denmark
[b] Department of Pathology, Aarhus University Hospital, 8000 Aarhus C, Denmark

Received 1 June 2006
Available online 2 October 2006

Abstract

Objective. Hydatidiform mole can be classified by histopathologic characteristics and by genetic constitutions and most complete moles are diploid, whereas most partial moles are triploid. We investigated the concordance between these two classifications, characterized moles with conflicting classifications, and compared the ability of the two classifications to discriminate between patients with and without a substantial risk of persistent trophoblastic disease.

Methods. 294 cases of consecutively collected hydropic placentas clinically suspected of hydatidiform mole made the basis of this retrospective study. We determined the ploidy and reviewed the original histopathologic material in all cases. Data on possible chemotherapy were collected for each patient.

Results. 270 of the conceptuses were histopathologically classified as hydatidiform mole. Among the 24 conceptuses classified as non-molar miscarriage, 20 were triploids, 2 were diploid androgenetic and 2 were diploid biparental. In 23% of the conceptuses, the histopathologic and genetic classifications were conflicting. 5% of the patients with hydropic placentas classified as partial mole encountered persistent trophoblastic disease; however, the genome was diploid in all these moles. None of 131 patients with a triploid hydropic gestation encountered persistent trophoblastic disease.

Conclusion. As full concordance between the histopathologic and the genetic classifications was not found, we believe that features beyond the genetic constitution influence the development of morphologic features in hydatidiform moles. We recommend that gestations suspected of hydatidiform mole are subjected to histopathologic examination. If hydatidiform change and trophoblastic hyperplasia are identified, the ploidy should be used to identify patients with a high risk of persistent trophoblastic disease.
© 2006 Elsevier Inc. All rights reserved.

Keywords: Hydatidiform mole; Persistent trophoblastic diseas; Ploidy

Introduction

Hydatidiform mole (HM) is an abnormal human gestation that is potentially malignant, as persistent trophoblastic disease (PTD) may follow. To identify those with PTD, patients are requested not to become pregnant during a surveillance period in which levels of hCG are monitored.

The classical criteria for the diagnosis of HM include swollen villi and trophoblastic hyperplasia [1] as two essential histopathologic hallmarks. Based on histopathologic observations, Vassilakos et al. [2] defined two classes of molar pregnancies: the complete and the partial mole. It is widely accepted that PTD is more frequent after a complete mole than after a partial mole, but patients with partial mole do have a considerable risk of PTD [3,4].

With the widespread use of ultrasonography in early pregnancy, abnormal conceptuses are identified earlier than before [5]. This may have introduced problems in the histopathologic classification of hydatidiform moles in that the morphologic characteristics are less developed within the first trimester [6,7].

Genetically, molar pregnancies are diploid, triploid or tetraploid, the latter being rare. Diploid moles are most often androgenetic [8,9] with two identical or different sets of paternal chromosomes, but a subset of diploid moles has a biparental genome [10]. Triploid moles have one maternal set of chromosomes and two paternal sets.

* Corresponding author. Fax: +45 89 49 43 70.
E-mail address: isani@as.aaa.dk (I. Niemann).

0090-8258/$ - see front matter © 2006 Elsevier Inc. All rights reserved.
doi:10.1016/j.ygyno.2006.08.025

Most often, but not always, diploid moles are classified as complete moles, whereas triploid moles most frequently are classified as partial [11].

We compared the histopathologic classification with the genetic classification of 294 hydropic placentas and characterized those with conflicting classifications. Furthermore, we assessed the ability of the two classification methods to identify subgroups of patients with and without a risk of PTD.

Methods

Material

Between April 1986 and June 2003, we received 386 unfixed representative samples of evacuated tissue from pregnancies clinically suspected of hydatidiform mole for genetic research analysis in the Danish Mole Project. Upon macroscopic examination, 309 samples containing at least 10 vesicles with a diameter >1 mm were classified as hydropic placentas. In eight of these 309 samples, we suspected a twin pregnancy due to the observation of fetal parts and clear demarcation between areas with normal and hydropic villi in the placental tissue. We excluded 77 samples that contained less than 10 vesicles with a diameter of >1 mm. From hospital files, pathology reports and data on gestational age and possible chemotherapy were collected for each patient. The Regional Committee on Biomedical Research Ethics of Aarhus County approved the study and all patients gave informed consent.

Genetic analyses

The ploidy of the 309 hydropic placentas was primarily determined by karyotyping before and/or after culture. After incubation, we counted five metaphases and karyotyped two, and after culture we counted 10 metaphases and karyotyped four. In 30 (10%) of the conceptuses, karyotyping failed. In 26 of these conceptuses and in 135 karyotyped conceptuses, we determined the nuclear DNA contents by flow cytometry. In the remaining four gestations, the ploidy was determined by analysis of DNA markers, see below. For flow cytometry, nuclei were isolated from unfixed tissue, stained with propidium iodide [12] and analyzed on a FACS Calibur (Becton Dickinson). Trout and chicken erythrocytes served as external controls. In all 135 conceptuses, the ploidies determined by karyotyping and by flow cytometry were identical.

The parental origin of the genome in the diploid hydropic placentas was assessed by analysis of polymorphic DNA markers. In brief, DNA was isolated from unfixed tissue and parental leucocytes using standard techniques. DNA was PCR amplified using a panel of ten primer pairs flanking microsatellite loci with a high heterozygosity. The PCR products were visualized using an ABI prism 310 Genetic Analyzer and sized with ABI prism GeneScan software (Applied Biosystems). The diploid conceptuses were classified as either homozygous androgenetic (identical paternal alleles), heterozygous androgenetic (different paternal alleles) or as biparental [13].

Among the 309 hydropic placentas, 151 were diploid androgenetic conceptuses, 14 were diploid biparental conceptuses, 131 were triploid, 5 were tetraploid and 8 were twin pregnancies with a diploid androgenetic conceptus and a normal diploid biparental fetus/placenta.

Histopathologic review

We succeeded in retrieving original histopathologic material from 294 of the 309 hydropic placentas. When the original slides were missing, new slides from the original paraffin blocks were reviewed. One gynecological pathologist (ESH) reviewed the 294 cases using the criteria given in the 2004 guidelines of the Danish Society of Gynecologists (Table 1) without knowing the genetic constitution or the original histopathologic classification of the conceptuses. "Unclassified mole" refers to gestations with vesicles and trophoblastic hyperplasia, but with too scarce or too degenerated material to classify it as either complete or partial mole.

Table 1
Histopathologic criteria for classification of hydatidiform mole as complete and partial mole [a]

	Macroscopic	Microscopic
Complete hydatidiform mole	• Vesicles 2–15 mm diffusely<br>• No fetal tissue	• Hydropic villi diffusely<br>• Trophoblastic hyperplasia frequent<br>• Stromal karyorrhetic debris<br>• No fetal tissue
Partial hydatidiform mole	• Vesicles 2–5 mm focally<br>• Possible fetal tissue	• Villi, normal and hydropic<br>• Trophoblastic hyperplasia focally<br>• Trophoblast pseudoinclusions<br>• Invaginated surface<br>• Fetal tissue frequent

[a] The 2004 criteria of the Danish Society of Gynecologists (www.dsog.dk/guidelines).

Statistical analysis

Statistical significance of differences between groups (χ^2 test) was determined in a Microsoft Excel 2000 spreadsheet.

Results

Histopathologic classification versus genetic classification

In the histopathologic review, 270 of the 294 hydropic placentas were classified as hydatidiform mole. Among the 24 hydropic placentas classified as non-molar miscarriages, 20 were triploids, two were diploid androgenetic and two were diploid biparental. As these two latter gestations neither met the morphologic criteria nor had the genetic constitution most often seen in hydatidiform mole, they most likely represented true non-molar hydropic abortions and were left out in the following.

In Table 2, the histopathologic classifications are correlated with the ploidy of 281 hydropic placentas (three tetraploid hydropic placentas and eight twin pregnancies are left out). Among the conceptuses classified as complete mole, 95% were diploid and 5% were triploid. Among the conceptuses classified as partial mole, 71% were triploid and 29% were diploid. With the genetic classifications as reference, 68% of the diploid hydropic placentas were classified as complete mole and 25% were classified as partial mole. Among the 12 diploid biparental hydropic placentas, nine were classified as partial mole and three as complete mole. Among the triploid hydropic placentas, 78% were classified as partial mole and 5% as complete mole.

By inspection of the unfixed samples, we suspected a twin pregnancy comprising one normal conceptus and one hydropic placenta in eight samples. By genetic analyses, we found that all eight conceptuses consisted of a diploid androgenetic hydropic placenta and normal diploid fetus with a normal placenta. The reviewing pathologist classified four of these eight conceptuses as twin pregnancies, while three were classified as partial mole, and one as an unclassified molar pregnancy.

In order to test the impact of the gestational age on the histopathologic classifications, we calculated the frequencies of

Table 2
Histopathologic and genetic diagnosis in 281 hydropic placentas

Genetic classification						
Histopathologic classification [a]	Diploid				Triploid	Total
	Androgenetic homozygous	Androgenetic heterozygous	Biparental	Genotype NA		
Complete mole	91	12	3		6	112
Partial mole	24	5	9	1	97	136
Hydatidiform mole, unclassified	9				2	11
Non-molar miscarriage	2				20	22
Total	126	17	12	1	125	281

NA: not analyzed.
[a] By the reviewing pathologist.

conceptuses with "false negative" histopathologic classification and stratified these by gestational age. "False negative" classification was defined as diploid androgenetic hydropic placentas classified as partial mole/non-molar miscarriage, diploid biparental hydropic placentas classified as partial mole, and triploid hydropic placentas classified as non-molar miscarriage. Leaving out 11 histopathologically unclassified moles and six triploid hydropic placentas classified as complete mole, 61 of 264 (23%) hydropic placentas had a "false negative" histopathologic classification (Table 3). "False negative" histopathologic classification was significantly more frequent among diploid than among triploid hydropic placentas ($p=0.04$). When stratifying the conceptuses by gestational age at evacuation, we observed a tendency towards a higher frequency of "false negative" classification among conceptuses evacuated after 1st trimester than within 1st trimester ($p=0.05$).

Persistent trophoblastic disease

Twenty-nine patients were subsequently treated with chemotherapy: 2 patients were diagnosed with choriocarcinoma, 18 with invasive mole and 9 patients were treated because of persistently elevated serum hCG levels (Table 4). All 29 conceptuses followed by PTD were classified as hydatidiform mole by both the primary and the reviewing pathologists. In four of these 29 moles, the primary and reviewing pathologists agreed on the classification as partial mole. In seven moles, either one or the other pathologist classified the mole as partial. With the classifications of the reviewing pathologist, 7 of 140 (5%) patients with partial mole encountered PTD. With the genetic classifications, none of the patients with triploid mole developed PTD, whereas 18% of the patients with diploid mole only achieved remission after chemotherapy ($p<0.001$). Two of the moles followed by PTD were diploid biparental moles (one classified as partial and one as complete mole). None of the 77 patients,

Table 3
"False negative"[a] histopathologic classifications relative to the genetic classifications in 264 hydropic placentas

Frequency of hydropic placentas with a "false" negative classification			
Ploidy	≤1st trimester ($n=150$)	>1st trimester ($n=114$)	Total
Diploidy ($n=147$)	21.4% (12/56)	31.9% (29/91)	27.9% (41/147)
Triploidy ($n=117$)	17.0% (16/94)	17.4% (4/23)	17.1% (20/117)
Total	18.7% (28/150)	28.9% (33/114)	23.1% (61/264)

[a] "False negative": diploid hydropic placentas classified as partial mole or non-molar miscarriages, and triploid hydropic placentas classified as non-molar miscarriage.

Table 4
Details on the moles in 29 patients subsequently treated with chemotherapy

Mole	Gestational age at evacuation (week)	Histopathologic diagnosis		Karyotype	Ploidy by flow cytometry	Parental origin of genome
		Primary pathologist	Reviewing pathologist			
325	15+1	CHM	CHM	46,XX	NA	P_1P_1
112	17+0	CHM	CHM	46	Diploid	P_1P_1
136	17+1	CHM	CHM	46,XX	Diploid	P_1P_1
180	15+4	CHM	CHM	46,XX	Diploid	P_1P_1
230	12+0	CHM	CHM	46,XX	Diploid	P_1P_1
454	9+3	PHM	CHM	46,XX	NA	P_1P_1
460	9+5	CHM	CHM	46,XY	NA	P_1P_2
563	10+5	CHM	CHM	46,XX	NA	P_1P_1
111	11+3	CHM	CHM	NA	Diploid	P_1P_1
497	19+4	CHM	CHM	46,XX	Diploid	PM
666	14+6	CHM	CHM	46,XX	NA	P_1P_1
409	11+3	CHM	CHM	46,XX	NA	P_1P_1
512	10+0	CHM	CHM	46,XX	Diploid	P_1P_1
437	11+6	CHM	CHM	46,XX	NA	P_1P_1
631	12+3	CHM	CHM	46,XX	NA	P_1P_1
605	10+1	CHM	CHM	46,XX	NA	P_1P_1
491	13+1	CHM	CHM	46,XX	NA	P_1P_1
427	9+5	CHM	CHM	46,XX	NA	P_1P_1
592	12+6	PHM	HM unclassified	46,XX	NA	P_1P_1
158	11+0	PHM	HM unclassified	46,XX	Diploid	P_1P_1
450 [a]	20+5	PHM	HM unclassified	46,XY	NA	P_1P_2
531	7+0	PHM	PHM	46,XY	NA	P_1P_2
550	13+6	PHM	PHM	46,XX	NA	P_1P_1
192	11+3	CHM	PHM	46,XX	Diploid	PM
594 [a]	6+4	PHM	PHM	46,XX	NA	P_1P_1
174	9+2	CHM	PHM	46,XX	Diploid	P_1P_2
443	19+2	CHM	PHM	46,XX	NA	P_1P_1
583	12+0	PHM	PHM	NA	NA	P_1P_1
235	9+5	PHM	NA	NA	Diploid	NA

CHM: complete hydatidiform mole, PHM: partial hydatidiform mole, HM: hydatidiform mole, NA: not analyzed.
P_1P_1: Androgenetic, homozygous. P_1P_2: Androgenetic, heterozygous. PM: Biparental.
[a] Twin pregnancy comprising diploid mole and normal fetus.

that were excluded because the sample submitted for genetic analyses lacked vesicles, developed PTD.

As a predictor for PTD among patients with HM, the sensitivity was 0.72 and the specificity was 0.58 for "complete mole". For "diploid mole," the sensitivity was 1.0 and the specificity was 0.50.

Discussion

We observed genetic heterogeneity among both complete and partial moles. In our study, ploidy was superior to morphology in discriminating between patients with a high and a low risk of PTD after a molar pregnancy.

One explanation of the inconsistency between morphology and ploidy could be related to the gestational age at evacuation. Recent studies have suggested that, in early moles, the hydropic change of the villi and the trophoblastic hyperplasia is less developed, making early complete moles resemble partial moles [14] and early partial moles mimic (non-molar) hydropic abortions [15]. The "false negative" classifications, however, were not more frequent in gestations evacuated within 1st trimester than later in pregnancy. Thus early evacuation does not explain the conflicting classifications in this study.

When comparing the histopathologic classifications with the genetic classifications, we observed that among the hydropic placentas classified as complete mole, the genome was diploid in 95% but triploid in 5%, whereas in the hydropic placentas classified as partial mole, the genome was triploid in 71% but diploid in 29%. Lage et al. [16] examined the ploidy in hydropic placentas and found that 79% of the placentas classified as complete mole were diploid and 21% were triploid, whereas 96% of the placentas classified as partial mole were triploid and 4% were diploid. In a study by Lawler et al. [17], 98% of the conceptuses classified as complete mole were diploid and 2% were triploid, whereas 98% of the conceptuses classified as partial mole were triploid and 2% were diploid. Although the concordance in the study by Lawler et al. is fairly high, none of the three studies demonstrated a universal concordance between the histopathologic and the genetic classifications. Varying interpretations of the histopathologic criteria may explain why agreement between the histopathologic and genetic classifications was not always reached. As seen in interobserver variation studies, disagreements between histopathologists are common [18]. Furthermore, one study demonstrated that interpretations of the histopathologic features changed when the histopathologists gained knowledge about the ploidy of the gestation [19].

Rather than looking for possible explanations as to why the classifications are not always concordant, we believe the disconcordancies between the histopathologic and the genetic classification indicate that different genetic constitutions can lead to identical morphology, and vice versa. It is possible that some of these discrepancies are caused by imperfect histopathologic criteria. It is also possible, however, that additional genetic subgroups are to be identified or that features beyond the genetic constitution influence the morphology of a molar pregnancy.

In order to gain knowledge about the biology of hydatidiform moles, we find it crucial to maintain both the histopathologic examination and the genetic analysis of pregnancies suspected of hydatidiform mole. By gathering and correlating the results obtained by these two methods, we will learn. If the histopathologic classification is "corrected" after addition of the results of ploidy determination or immunohistochemical assessment of expression of genes subjected to parental imprinting [20], we will loose information.

Using "complete mole" as a predictor of risk of PTD raises clinical problems because a fraction of the moles with a malignant potential are classified as partial moles. In the present study and in other studies [3,4], up to 5% of the patients with partial moles received chemotherapy, and like others [21,22] we classified conceptuses with all different genetic constitutions as partial mole. Three pregnancies with a normal twin and a diploid androgenetic mole were classified as partial mole. Such pregnancies have a 20% risk of PTD and must not be misclassified [23]. Another clinical problem concerns the number of moles that are not recognized as such with the current histopathologic criteria. In the present study, 16% of the triploid hydropic placentas and 1% of the diploid androgenetic hydropic placentas were classified as non-molar miscarriages. However, as the triploid moles have an exceedingly low risk of PTD [13], these "false negatives" may not have profound clinical consequences.

In our study group, all patients with PTD had a diploid molar pregnancy (including two patients with twin pregnancies) ($p<0.001$). Compared with complete mole, diploid mole was just as specific but was a more sensitive predictor of PTD.

For clinical use, instead of complicating the diagnostic procedures by implying more detailed histopathologic criteria [7,24,25], we suggest a simplification: histopathologic examination should be performed to identify gestations with edematous villi and trophoblastic hyperplasia among miscarriages. Ploidy should be used as a predictor for subsequent PTD or spontaneous remission, respectively, after a molar pregnancy.

Acknowledgments

This study was supported by the Research Initiative of Aarhus University Hospital and the "Frits, Georg og Marie Cecilie Gluds Legat".

References

[1] Szulman AE, Surti U. The syndromes of hydatidiform mole: I. Cytogenetic and morphologic correlations. Am J Obstet Gynecol 1978;131:665–71.

[2] Vassilakos P, Riotton G, Kajii T. Hydatidiform mole: two entities. A morphologic and cytogenetic study with some clinical consideration. Am J Obstet Gynecol 1977;127:167–70.

[3] Goto S, Yamada A, Ishizuka T, Tomoda Y. Development of postmolar trophoblastic disease after partial molar pregnancy. Gynecol Oncol 1993; 48:165–70.

[4] Feltmate CM, Batorfi J, Fulop V, Goldstein DP, Doszpod J, Berkowitz RS. Human chorionic gonadotropin follow-up in patients with molar pregnancy: a time for reevaluation. Obstet Gynecol 2003;101:732–6.

[5] Sebire NJ, Rees H, Paradinas F, Seckl M, Newlands E. The diagnostic implications of routine ultrasound examination in histologically confirmed early molar pregnancies. Ultrasound Obstet Gynecol 2001;18:662–5.

[6] Hui P, Martel M, Parkash V. Gestational trophoblastic diseases: recent advances in histopathologic diagnosis and related genetic aspects. Adv Anat Pathol 2005;12:116–25.

[7] Keep D, Zaragoza MV, Hassold T, Redline RW. Very early complete hydatidiform mole. Hum Pathol 1996;27:708–13.
[8] Kajii T, Ohama K. Androgenetic origin of hydatidiform mole. Nature 1977;268:633–4.
[9] Ohama K, Kajii T, Okamoto E, Fukuda Y, Imaizumi K, Tsukahara M, et al. Dispermic origin of XY hydatidiform moles. Nature 1981;292:551–2.
[10] Kovacs BW, Shahbahrami B, Tast DE, Curtin JP. Molecular genetic analysis of complete hydatidiform moles. Cancer Genet Cytogenet 1991; 54:143–52.
[11] Lawler SD, Pickthall VJ, Fisher RA, Povey S, Evans MW, Szulman AE. Genetic studies of complete and partial hydatidiform moles. Lancet 1979;2:580.
[12] Vindelov LL, Christensen IJ, Nissen NI. A detergent-trypsin method for the preparation of nuclei for flow cytometric DNA analysis. Cytometry 1983;3:323–7.
[13] Niemann I, Petersen LK, Hansen ES, Sunde L. Predictors of low risk of persistent trophoblastic disease in molar pregnancies. Obstet Gynecol 2006;107:1006–11.
[14] Mosher R, Goldstein DP, Berkowitz R, Bernstein M, Genest DR. Complete hydatidiform mole. Comparison of clinicopathologic features, current and past. J Reprod Med 1998;43:21–7.
[15] Genest DR. Partial hydatidiform mole: clinicopathological features, differential diagnosis, ploidy and molecular studies, and gold standards for diagnosis. Int J Gynecol Pathol 2001;20:315–22.
[16] Lage JM, Mark SD, Roberts DJ, Goldstein DP, Bernstein MR, Berkowitz RS. A flow cytometric study of 137 fresh hydropic placentas: correlation between types of hydatidiform moles and nuclear DNA ploidy. Obstet Gynecol 1992;79:403–10.
[17] Lawler SD, Fisher RA, Dent J. A prospective genetic study of complete and partial hydatidiform moles. Am J Obstet Gynecol 1991;164:1270–7.
[18] Howat AJ, Beck S, Fox H, Harris SC, Hill AS, Nicholson CM, et al. Can histopathologists reliably diagnose molar pregnancy? J Clin Pathol 1993; 46:599–602.
[19] Fukunaga M, Katabuchi H, Nagasaka T, Mikami Y, Minamiguchi S, Lage JM. Interobserver and intraobserver variability in the diagnosis of hydatidiform mole. Am J Surg Pathol 2005;29:942–7.
[20] Castrillon DH, Sun D, Weremowicz S, Fisher RA, Crum CP, Genest DR. Discrimination of complete hydatidiform mole from its mimics by immunohistochemistry of the paternally imprinted gene product p57KIP2. Am J Surg Pathol 2001;25:1225–30.
[21] Genest DR, Ruiz RE, Weremowicz S, Berkowitz RS, Goldstein DP, Dorfman DM. Do nontriploid partial hydatidiform moles exist? A histologic and flow cytometric reevaluation of nontriploid specimens. J Reprod Med 2002;47:363–8.
[22] Ohama K, Ueda K, Okamoto E, Takenaka M, Fujiwara A. Cytogenetic and clinicopathologic studies of partial moles. Obstet Gynecol 1986; 68:259–62.
[23] Sebire NJ, Foskett M, Paradinas FJ, Fisher RA, Francis RJ, Short D, et al. Outcome of twin pregnancies with complete hydatidiform mole and healthy co-twin. Lancet 2002;359:2165–6.
[24] Sebire NJ, Fisher RA, Rees HC. Histopathological diagnosis of partial and complete hydatidiform mole in the first trimester of pregnancy. Pediatr Dev Pathol 2003;6:69–77.
[25] Sebire NJ, Makrydimas G, Agnantis NJ, Zagorianakou N, Rees H, Fisher RA. Updated diagnostic criteria for partial and complete hydatidiform moles in early pregnancy. Anticancer Res 2003;23:1723–8.
