# Supplementary material for: Triploidy—Observations in 154 Diandric Cases
Source: PLoS One. 2015 Nov 12;10(11):e0142545. doi: 10.1371/journal.pone.0142545 (PMC4642992; doi:10.1371/journal.pone.0142545)
Supplement: S1 Table — Identical twins are 100% identical by descent (PI_HAT = 1.0), first-degree relatives are on average 50% IBD (PI_HAT = 0.5), second-degree relatives are on average 25% IBD (PI_HAT = 0.25). For each comparison, Z2, Z1 and Z0 are the proportion of markers showing 2, 1 and 0 alleles identical by descent, respectively. The IDB proportions and PI_HAT showed that the mother has no genetic relationship with the molar pregnancy. The normal conceptus and the mole were related as parent-child (and not as siblings) because they share the paternal genome identical by descent. (DOCX) [file pone.0142545.s003.docx]

**S1 Table**

| **Sample 1** | **Sample 2** | **Z0** | **Z1** | **Z2** | **PI_HAT** |
| --- | --- | --- | --- | --- | --- |
| C0301q (mother) | C0301B (mole) | 1 | 0 | 0 | 0 |
| C0301q (mother) | C0301V (twin normal conceptus) | 0 | 1 | 0 | 0,5 |
| C0301B (mole) | C0301V (twin normal conceptus) | 0 | 1 | 0 | 0,5 |
|  |  |  |  |  |  |
| **Expected (listed for comparison)** | |  |  |  |  |
| Parent | Child | 0 | 1 | 0 | 0,5 |
| Sibling1 | Sibling2 | 0,25 | 0,5 | 0,25 | 0,5 |
